# Deficiency of STING Signaling in Embryonic Cerebral Cortex Leads to Neurogenic Abnormalities and Autistic‐Like Behaviors

**DOI:** 10.1002/advs.202002117

**Published:** 2020-11-03

**Authors:** Dongming Zhang, Chang Liu, Hong Li, Jianwei Jiao

**Affiliations:** ^1^ State Key Laboratory of Stem Cell and Reproductive Biology Institute of Zoology Chinese Academy of Sciences Beijing 100101 China; ^2^ Medical School University of Chinese Academy of Sciences Beijing 100049 China; ^3^ Academy for Advanced Interdisciplinary Studies Peking University Beijing 100871 China; ^4^ Innovation Academy for Stem Cell and Regeneration Beijing 100101 China

**Keywords:** autistic‐like behavior, brain development, NF‐*κ*B, STING

## Abstract

STING is known as a central adaptor for sensing cytosolic DNA sensing. Recent studies have provided evidence that STING response is divergent among different cell types. Here, this work demonstrates that STING controls neural progenitor cells (NPCs) by sensing DNA damage in NPCs. The deletion of STING reduces neuronal differentiation and increases proliferation of mouse and human NPCs. Furthermore, STING^cKO^ mice display autistic‐like behaviors. In NPCs, STING specifically recruits IKK*β* and activates nuclear factor *κ*B (NF‐*κ*B) through phosphorylation. NF‐*κ*B binds to ALX4 promoter and triggers ALX4 transcription. In addition, tumor necrosis factor *α*, an activator of NF‐*κ*B, can rescue some phenotypes caused by STING deletion in mice. Together, the findings show that STING signaling is essential for neuronal gene expression program and has profound consequences on brain function.

## Graphical abstract

DNA damage–STING signaling pathway plays an important role for differentiation and proliferation of neural progenitors. Ablation of STING influences neurogenesis both in mouse and human neural progenitors. Mice loss of STING in brain display autistic‐like behaviors. Mechanistically, STING can activate nuclear factor *κ*B pathway to regulate ALX4 expression. ALX4 is an important downstream gene of STING during embryonic neurogenesis.

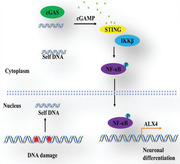

## 1 Introduction

Cerebral cortex development is a temporally and spatially regulated process that is defined by an early expansion of proliferative neural progenitor cells (NPCs), which are mainly responsible for self‐renewal, the differentiation of neurons, and the generation of postmitotic neurons.^[^ ^1^, ^2^ ^]^ During the brain development, each process is accurately regulated by complex interactions between genes and extrinsic signals and any abnormal stimuli are likely to change the fate of NPCs and then affect function of the brain.^[^ ^3^ ^]^ DNA double strand breaks (DSBs) in cells frequently happen under exogenous and endogenous events, which are essential for some normal physiological functions.^[^ ^4^, ^5^ ^]^ For proliferative cells, DNA replication is a main endogenous source of DNA breakage.^[^ ^6^ ^]^ Several studies suggest that DSBs‐initiated p53 signaling can promote NPCs differentiation.^[^ ^7^, ^8^ ^]^ However, it is still largely unknown how DSBs‐initiated molecular pathways regulate neuronal differentiation.

Recently, many studies have demonstrated that the STING signaling pathway could be activated by DNA damage.^[^ ^9^, ^10^, ^11^, ^12^, ^13^, ^14^, ^15^ ^]^ STING, stimulator of IFN genes, is an important cytoplasmic pattern recognition receptor, which can be activated by the accumulation of DNA in the cytoplasm.^[^ ^16^, ^17^ ^]^ STING is mainly localized in the endoplasmic reticulum (ER). Following its activation, STING traffics from the ER to perinuclear signaling compartments, where STING recruits the kinase TBK1 or IKK*β*, which mediate the activation of the transcription factor interferon regulatory factor 3 (IRF3) or nuclear factor *κ*B (NF‐*κ*B).^[^ ^18^, ^19^, ^20^ ^]^ Then the activated IRF3 or NF‐*κ*B transports into nucleus to regulate gene expression. In addition, cGAS is a well‐studied DNA‐binding protein, which can produce cGAMP, a second messenger, to activate downstream adaptor molecule STING.^[^ ^21^ ^]^ Besides its main function in eliciting effective immunoreaction against microbial pathogens, STING signaling also plays an important role in various cell types, such as cardiomyocytes,^[^ ^22^, ^23^ ^]^ intestinal epithelium,^[^ ^24^ ^]^ and cancer cell.^[^ ^15^, ^25^ ^]^ However, the role of STING signaling in NPCs during brain development has never been reported.

In this study, we found that *γ*H2AX, DNA damage marker, were highly expressed in NPCs during embryonic cerebral cortex development and the expression of STING was highly correlated with *γ*H2AX. The deletion of STING in embryonic brain promotes the proliferation of NPCs and inhibits neuronal differentiation. Neurons in STING conditional knockout (KO) (STING^cKO^) mice display abnormal dendrites. And the abnormal brain development results in mice autistic‐like behaviors. Mechanistically, STING regulates the expression of ALX4 by increasing NF‐*κ*B phosphorylation to promote NPCs differentiation. We also discovered that tumor necrosis factor *α* (TNF‐*α*) could rescue some phenotypes in STING^cKO^ mice. In addition, STING knockout human NPCs or cortical organoids exhibited neuronal differentiation delay. Together, our data provide a new insight into the roles of STING signaling during brain development.

## 2 Results

### 2.1 DNA Damage Induces STING Expression in the Early Neuronal Progenitor Cells

**Figure 1:**
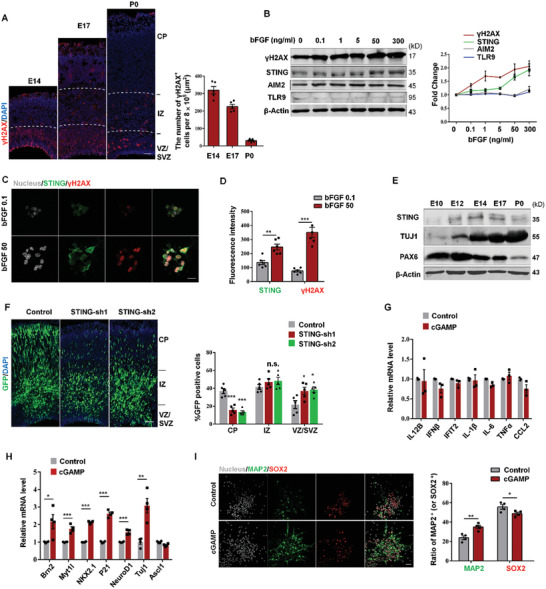
STING is correlated with DNA damage and affects neural progenitor cells. A) Images of cerebral cortex sections of E14, E17, and P0 labeled for *γ*H2AX and DAPI. The bar graph shows the number of *γ*H2AX^+^ cells in the VZ/SVZ per 8 × 10^5^ µm^2^. SVZ, subventricular zone; VZ, ventricular zone; IZ, intermediate zone; CP, cortical plate. Scale bar, 100 µm. B) Western blot analysis of *γ*H2AX, STING, AIM2, and TLR9 in neural progenitor cells (NPCs) after treatment with bFGF of various concentrations for 16 h in proliferation medium (suspension culture) (*n* = 4 independent experiments). C,D) NPCs were isolated at E12.5 and then treated with bFGF (0.1, 50 ng mL^−1^) for 16 h in proliferation medium (suspension culture). Then cells were fixed and costained with anti‐*γ*H2AX and anti‐STING antibodies. Scale bar, 15 µm (C). D) Right, bar graph shows the fluorescence intensity of STING and *γ*H2AX (*n* = 6 field of view of three individual experiments). E) STING is expressed in the development of the cerebral cortex. Cerebral cortex of different developmental stages (E10, E12, E14, E17, and P0) were isolated and lysed for Western blot analysis of STING, Tuj1, PAX6 (*n* = 3 individual experiments). F) STING knockdown results in abnormal cell position in embryonic cerebral cortex. Left, STING knockdown or control plasmids were electroporated into brain of E13.5 and analyzed at E16.5. Right, bar graph shows the percentage of GFP‐positive cells in each region (*n* = 5 embryos from three different mothers). G) NPCs from E12.5 cerebral cortex were treated with 0.2 × 10^−6^ m cGAMP for 1 d before qRT‐PCR analysis of neural differentiation‐related gene mRNA and H) innate immune‐related gene mRNA levels. I) NPCs were isolated from E12.5 cerebral cortex and cultured for 2 d. Then cells were treated with 0.2 × 10^−6^ m cGAMP for 1 d before fixed and costained with anti‐SOX2 and anti‐MAP2 antibodies. Bar graph shows the ratio of MAP2 or SOX2‐positive cells in control and cGAMP treated groups (*n* = 4 independent experiments). Scale bar, 30 µm. Error bars represent means ± SEM; two‐tailed unpaired *t*‐test, n.s., not significant, *P < 0.05, **P < 0.01, or ***P < 0.001.

Previous studies have reported that DNA damage occurs in some stem cells, such as embryonic stem cells^[^ ^26^ ^]^ and hematopoietic stem cells,^[^ ^27^ ^]^ however, for neuronal progenitor cells is unclear. *γ*H2AX, a sensitive marker of DNA damage, was expressed predominantly in the ventricular zone (VZ)/subventricular zone (SVZ) and was highly expressed at E14 and gradually decreased with the progression of development (**Figure** **1**A and Figure S1A, Supporting Information). Basic fibroblast growth factor (bFGF) is a well‐known neurotrophic factor for proliferating NPC.^[^ ^28^, ^29^, ^30^ ^]^ To further determine whether DNA damage exists mainly in rapidly proliferating NPCs, we cultured primary NPCs in proliferation medium with different concentrations of bFGF. The phosphorylation levels of histone *γ*H2AX were correlated with the concentrations of bFGF (Figure 1B). In addition, we found that *γ*H2AX was increased in the nucleus of NPCs derived from E12 cerebral cortex after treatment with cytosine *β*‐d‐arabinofuranoside hydrochloride (Ara‐C) which is a genotoxic replication inhibitor (Figure S1B, Supporting Information). In addition, more cytosolic DNA were detected in NPCs after treatment with two DNA damaging agents (DDA) (Figure S1C, Supporting Information). Thus, we screened three main DNA‐activated molecules:^[^ ^31^ ^]^ STING, AIM2, and TLR9. And we found the expression of STING was correlated with *γ*H2AX in NPCs (Figure 1B–D), indicating that STING might have important roles in NPCs.

To explore the effects of STING in NPCs, we first detected STING expression in embryonic cortex from E10 to P0. We found that STING was highly expressed at E14 and gradually decreased from E14 to P0, and the expression of the progenitor markers PAX6 decreased but the primary neuron marker TUJ1 increased during this period (Figure 1E). Immunostaining revealed that STING was mainly expressed in the VZ/SVZ in the early embryonic cortex (Figure S2A, Supporting Information). In vitro, we observed that STING was coexpressed with SOX2 and NESTIN in primary NPCs (Figure S2B, Supporting Information). To further study whether STING plays a unique function in neurogenesis during brain development. We generated two highly effective targeting STING shRNA plasmids. Exogenous or endogenous STING was obviously reduced in 293A or primary NPCs by STING‐shRNA (Figure S2C,D, Supporting Information). We used in utero electroporation (IUE) to introduce STING‐shRNA into NPCs in E13 embryonic cortex in pregnant mice. Interestingly, we discovered that knockdown of STING led to significant abnormal distribution of GFP‐positive cells at E16. There were more GFP‐positive cells in the proliferation VZ/SVZ, and GFP‐positive cells were significant reduced in the cortical plate (CP) (Figure 1F). To confirm whether STING knockdown results in abnormal neuronal development, we immunostained electroporated cortex sections with neuronal marker TUJ1 or progenitor markers PAX6 and TBR2. We found that the percentage of GFP‐TUJ1 double‐positive cells was decreased in CP, and GFP‐PAX6 or GFP‐TBR2 double‐positive cells were increased in VZ/SVZ. Western blot results also showed the increased protein level of PAX6 and decreased level of TUJ1 in vitro (Figure S2D, Supporting Information). These results demonstrate that STING is required for cortical neurogenesis.

cGAS was a well‐studied upstream molecule of STING. The cGAMP synthesized by cGAS can activate the adaptor STING.^[^ ^21^ ^]^ Knockdown of cGAS in NPCs reduced STING expression (Figure S3A,B, Supporting Information). We also investigate the role of cGAS in NPCs. Similarly, when the expression of cGAS was downregulated, the GFP cells in the VZ/SVZ were increased, and GFP cells in the CP were obviously reduced (Figure S3C, Supporting Information). While exogenous overexpression of cGAS results in significant increased GFP cells in CP and decreased GFP cells in VZ/SVZ (Figure S3D, Supporting Information). In vitro, after treatment of 0.2 × 10^−6^ m cGAMP, we detected that the mRNA levels of several innate immune‐related cytokines had no significant change in NPCs (Figure 1G). However, the expression of some neural differentiation related genes was obviously increased (Figure 1H). Immunostaining results also showed that cGAMP inhibited the proliferation of NPCs and promoted differentiation into neurons (Figure 1I). Together, these results suggest that STING signaling is required for cortical neurogenesis.

### 2.2 Loss of STING Expression Impairs Early Neurogenesis

**Figure 2:**
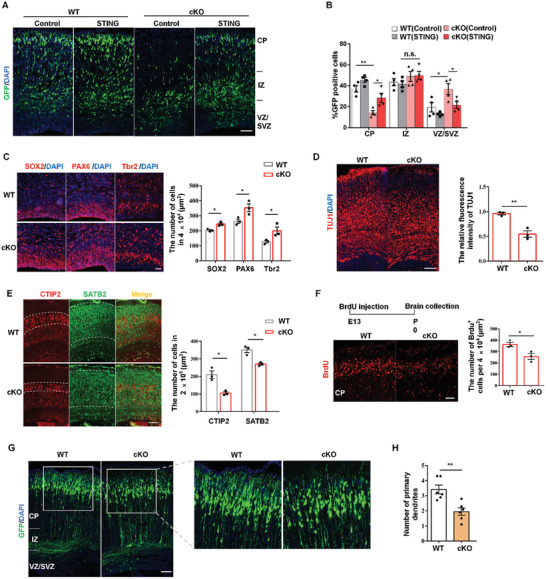
STING deletion in brain impairs neurogenesis. A) STING overexpression or control plasmid was electroporated into STING^fl/fl^ or STING^cKO^ mice brain of E13 and analyzed at E16 (*n* = 4 brains). B) Bar graph shows the quantification of the percentage of GFP^+^ cells in different regions of cortex. Scale bar: 100 µm. C) Brain sections of STING^fl/fl^ and STING^cKO^ mice at E16 were immunostained, respectively, for NPCs markers SOX2, PAX6, and Tbr2 with DAPI. Bar graph shows the number of SOX2 or PAX6 or Tbr2 cells in the VZ/SVZ per 40 000 µm^2^ (*n* = 3 brains). Scale bar: 50 µm. D) The ratio of TUJ1^+^ cells was reduced in STING^cKO^ mice. STING^fl/fl^ and STING^cKO^ mice brains were collected and immunostained by anti‐TUJ1 antibody at P0. Bar graph shows the relative fluorescence intensity of TUJ1 (*n* = 3 brains). Scale bar: 100 µm. E) P0 brain sections of STING^fl/fl^ and STING^cKO^ mice were costained with anti‐SATB2 and anti‐CTIP2 antibodies. Bar graph shows the number of SATB2^+^ (or CTIP2^+^) cells per 250 000 µm^2^. Scale bar: 100 µm. F) BrdU (100 mg kg^−1^) was intraperitoneal injected at E13, Then, STING^fl/fl^ and STING^cKO^ mice brains were collected for immunostaining analysis using anti‐BrdU antibody at P0. Bar graph shows the number of BrdU^+^ cells in CP per 40 000 µm^2^ (*n* = 3 brains). Scale bar: 50 µm. G) IUE was performed in the STING^fl/fl^ or STING^cKO^ mice at E13.5 and analyzed at P0. GFP‐positive cells show that STING knockout has abnormal leading processes compared to the WT. Scale bar, 100 µm. H) Quantification of primary dendritic numbers in STING^fl/fl^ and STING^cKO^ cortices (*n* = 6 brains). Error bars represent means ± SEM; two‐tailed unpaired *t*‐test, n.s., not significant, *P < 0.05, **P < 0.01, or ***P < 0.001.

To further investigate the role of STING in whole cortical development, we bred the STING^fl/fl^ mice with Nestin‐Cre mice to generate STING conditional knockout (STING^cKO^) mice (Figure S4A, Supporting Information). Then, we performed IUE with GFP plasmid of STING^fl/fl^ mice and STING^cKO^ mice at E13. Compared to STING^fl/fl^ mice, STING^cKO^ mice showed an abnormal GFP cells distribution at E16 (Figure S4B, Supporting Information), which was similar to the phenotype of STING knockdown. We also found that overexpression of STING in STING^cKO^ mice caused the percentage of GFP‐positive cells decreased in the VZ/SVZ and increased in CP compared to controls (**Figure** **2**A,B). Further, Western blot results showed that PAX6 or TUJ1, two neurogenesis‐associated markers, were changed when STING was deleted in cortex (Figure S4C, Supporting Information).

Immunostaining for STING^cKO^ brain at E16 showed that the progenitor markers of SOX2, PAX6, and TBR2 were increased (Figure 2C). And neuronal markers of TUJ1, STAB2, and CTIP2 were decreased at P0 (Figure 2D,E). In addition, we found that STING^cKO^ did not show significant apoptosis in cerebral cortex (Figure S4D, Supporting Information). To further investigate the effect of STING on terminal mitosis of NPCs and neuronal differentiation, BrdU birthdating experiment was performed.^[^ ^32^ ^]^ BrdU was intraperitoneally injected at E13, and wild‐type (WT) and STING^cKO^ brains were collected at P0. Immunostaining of BrdU showed that BrdU^+^ cells were obviously decreased in STING^cKO^ group, indicating that STING can promote NPCs terminal mitosis and facilitate neural differentiation (Figure 2F).

We also performed long‐term IUE experiment from E13 to P0 with GFP plasmids in WT and STING^cKO^ mice. The results showed that WT and STING^cKO^ GFP cells almost migrated to upper layer of cerebral cortex (Figure 2G). However, we found that the STING‐loss neurons exhibited abnormal morphology (Figure 2G,H). In order to further explore the influence of STING deletion on neuronal morphology, primary NPCs were isolated from electroporated brains. The results showed that the total length of the dendrites was reduced in STING‐deleted neurons after 3 d culture (Figure S4E,F, Supporting Information). Altogether, these results demonstrate that STING is essential for neuronal development and STING deletion leads to abnormal neuronal development.

### 2.3 Loss of STING Leads to Autistic‐Like Behaviors in Adult Mice

**Figure 3:**
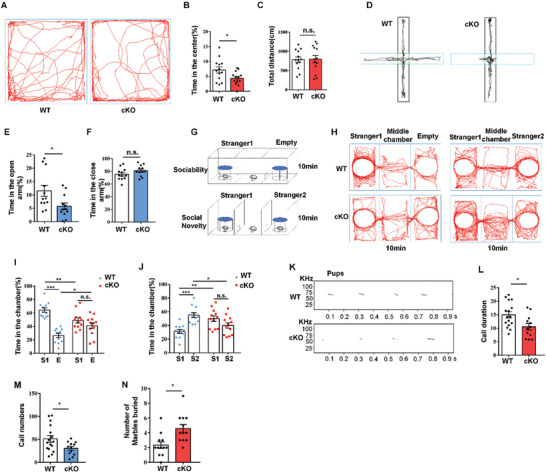
The loss of STING results in autistic‐like behaviors in mice. A) Representative image of tracing pathway of STING^fl/fl^ and STING^cKO^ mice in the open field test. B) Time in the center was reduced in STING^cKO^ mice. C) The traveling distance was not different between STING^fl/fl^ and STING^cKO^ mice within 5 min. D) Representative tracks in the elevated‐plus maze test. E,F) Time spent in the open and closed arms. G) The model of the sociability and social novelty test. H) Representative tracks from “Stranger 1‐Empty” and “Stranger 1‐Stranger 2” of STING^fl/fl^ and STING^cKO^ mice. I) STING^cKO^ mice spent less time in the left chamber (Stranger 1) and more time in the right chamber (empty cage) when compared with STING^fl/fl^. J) STING^cKO^ mice spent less time for the novel partner (Stranger 2) and spent more time for Stranger 1 when compared with STING^fl/fl^. K) Representative USVs spectrogram in isolated pups. L,M) Significant difference of call duration and call numbers in USVs between isolated STING^fl/fl^ and STING^cKO^ pups (*n* = 16 (WT), *n* = 12(cKO)). N) STING^cKO^ mice bury more marbles significantly than WT mice. *n* = 12 wild‐type mice and 12 STING^cKO^ mice. Error bars represent means ± SEM; *P* values were calculated by one‐way ANOVA with Tukey's post hoc, n.s., not significant, *P < 0.05, **P < 0.01, or ***P < 0.001.

STING knockout in NPCs disrupts the neurogenesis during the embryonic development. However, the brain weights of P0 and adult STING^cKO^ mice were indistinguishable from those of wild‐type mice (Figure S5A,B, Supporting Information). There are also no significant difference in bodyweight between cKO and wild type (Figure S5C, Supporting Information). Then, we wondered whether STING^cKO^ mice have any behavioral defects. STING^cKO^ mice and their littermate WT mice were first tested in open field. The STING^cKO^ mice spent less time in the central area, although the traveled distance had no apparent difference (**Figure** **3**A–C). Next, we performed elevated plus‐maze test, the result showed that STING^cKO^ mice spent less time in the open arms (Figure 3D–F). The result indicated that STING^cKO^ mice showed exploration deficiency. Then, the forced swim test and Y‐maze test were performed to detect whether STING^cKO^ mice had depressive‐like states and working memory deficit. However, we found that there was no difference in the forced swim test or Y‐maze test between WT and STING^cKO^ mice (Figure S5D–F, Supporting Information). To determine whether STING^cKO^ mice displayed unusual social interaction, we performed three‐chamber test (Figure 3G). STING^cKO^ mice showed less interest for exploring a strange mouse‐containing cage (Stranger 1) over an empty cage (Figure 3H,I). Then, when a novel mouse (Stranger 2) was placed in another empty cage, STING^cKO^ mice showed no significant interest for exploring the strange 2 mouse (Figure 3H,J). These results suggest that STING deletion disrupted mouse social interaction abilities. Thus, we speculated that STING knockout in brain might leads to autistic‐like behavior. Then, we recorded ultrasonic vocalization (USV) on pups at P5. STING^cKO^ pups produced fewer calls and shorter call durations than wild‐type pups (Figure 3K–M). We also observed that STING^cKO^ mice had more digging than WT mice in marble burying test (Figure 3N and Figure S5G, Supporting Information). Taken together, these results suggest that STING^cKO^ mice display autism‐related behaviors.

### 2.4 STING Regulates Neurogenesis through Targeting the Transcriptional Factor ALX4

**Figure 4:**
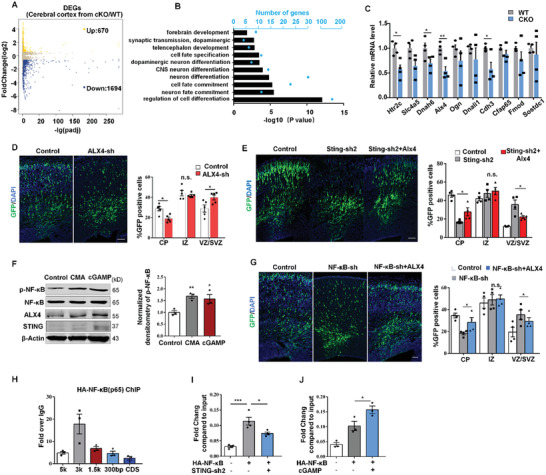
STING regulates neurogenesis by targeting Alx4 through NF‐*κ*B pathway. A) Differentially expressed genes (DEGs) were identified from E13 forebrain of WT and cKO mice. Yellow dots represent upregulated DEGs. Blue dots represent downregulated DEGs. B) Analysis of gene ontology of biological functions for DEGs in STING‐depleted E13 forebrain. C) List of top ten genes, which are downregulated, were detected by qPCR in WT and cKO groups at E13 (*n* = 4). D) Control or Alx4‐sh plasmids were electroporated into the brains at E13, and then the brains were harvested at E16. Bar graph shows the percentage of GFP cells in the CP, IZ, and VZ/SVZ (*n* = 5 brains). Scale bar, 100 µm. E) STING knockdown caused abnormal GFP‐positive cell distribution in the embryonic brain, and the Alx4 overexpression could rescue this phenotype partially (*n* = 4 brains). F) NPCs were treated with 50 µg mL^−1^ 10‐carboxymethyl‐9‐acridanone (CMA) or 0.2 × 10^−6^ m cGAMP for 1 d in proliferation medium. p‐NF‐*κ*B(p65), NF‐*κ*B (p65), ALX4, and STING protein levels were analyzed (*n* = 3 independent experiments). G) NF‐*κ*B (p65) knockdown caused abnormal GFP cell position in embryonic brain, and Alx4 overexpression could partially rescue this phenotype (*n* = 4 brains). H) NF‐*κ*B (p65) binding enrichment on indicated regions of Alx4 gene revealed by ChIP‐qPCR (*n* = 3 independent chromatin samples). I) The enrichment of NF‐*κ*B (p65) on promoters of Alx4 were analyzed with or without STING‐sh2 in NPCs (*n* = 4 independent chromatin samples). J) The enrichment of NF‐*κ*B (p65) on promoters of Alx4 in NPCs were analyzed with or without cGAMP (*n* = 3 independent chromatin samples). Error bars represent means ± SEM; two‐tailed unpaired *t*‐test, n.s., not significant, *P < 0.05, **P < 0.01, or ***P < 0.001.

To investigate molecular mechanisms associated with the phenotypes of STING deletion, we examined global transcriptome by RNA sequencing (RNA‐seq) analysis in E13 cerebral cortex. Altogether, the most differentially expressed genes (DEGs) were downregulated in STING^cKO^ samples (**Figure** **4**A and Figure S6A, Supporting Information). Gene ontology (GO) term analysis showed that a lot of DEGs were enriched in biological processes related to neuronal fate commitment (Figure 4B). To investigate how deletion of STING influences the molecular program, we first verified the expression levels of top ten most obviously downregulated genes by RT‐PCR. We noticed that ALX4, a transcriptional factor, was greatly reduced (Figure 4C and Figure S6B, Supporting Information). We also confirmed that STING deletion reduced ALX4 protein levels at E16 in the cortex (Figure S6E, Supporting Information). We used IUE with knockdown plasmids to study the effects of ALX4 and the other three obviously downregulated genes (Htr2c, Dnah6, and Slc4a5) from E13 to E16. The results showed that only Alx4 had a similar phenotype with STING (Figure 4D and Figure S6C,D, Supporting Information). ALX4‐overexpression plasmids were coelectroporated with Sting‐shRNA into E13 brains, and harvested at E16. We found that coexpression of ALX4 with Sting‐sh2 can partly rescue the abnormal distribution of GFP‐positive cells (Figure 4E). In addition, CMA and cGAMP, two STING activators, could obviously promote expression of ALX4 and STING (Figure 4F). These results suggest that ALX4 is a downstream gene of STING during embryonic neurogenesis. To further explore the role of ALX4 in cortical development, we detected protein levels of ALX4 in cerebral tissue from E12 to P0. ALX4 was highly expressed at E14 during cortical development, which was similar to STING (Figure S6F, Supporting Information). We examined ALX4 protein in the developing cerebral cortex (E13, E16). ALX4 is widely expressed in embryonic cortex and coexpressed with SOX2 in the VZ/SVZ or TUJ1 in the CP (Figure S6G, Supporting Information). In vitro, ALX4 was also mainly coexpressed with Tuj1 (Figure S6H, Supporting Information). Electroporated cortex sections were immunostained with progenitor markers (SOX2 and TBR2). We found that with the knockdown of ALX4, the percentage of GFP‐SOX2 or GFP‐TBR2 double‐positive cells was increased in VZ/SVZ (Figure S6I,J, Supporting Information). In summary, ALX4 plays consistent roles with STING in embryonic neurogenesis.

### 2.5 STING Regulates ALX4 Expression by NF‐*κ*B Signaling Pathway

To explore how STING regulates ALX4 expression, we screened several candidates, including NF‐*κ*B, TBK1, IRF3, IKK*β*, and STAT6, which can interact with STING and regulate gene expression as previous studies reported.^[^ ^33^, ^34^ ^]^ We found that phosphorylated NF‐*κ*B (p65) was significantly downregulated in STING deleted cortex (Figure S7A, Supporting Information). The phosphorylation level of NF‐*κ*B (p65) is also dynamically changed during cortex development (Figure S7E, Supporting Information). Next, we constructed the knockdown plasmids for each candidate. IUE results showed that NF‐*κ*B and IKK*β* knockdown led to abnormal GFP‐positive cells distribution compared with control (Figure S7C,D,F, Supporting Information). We also detected that phosphorylation level of NF‐*κ*B increased by the treatment of CMA and cGAMP (Figure 4F). However, ablation of NF‐*κ*B did not influence the expression of STING (Figure S7F, Supporting Information). Previous study demonstrated that NF‐*κ*B can be activated by the IKK complex and that IKK*β* was critical for gene expression via NF‐*κ*B activation.^[^ ^35^ ^]^ Thus, we speculated that STING might interact with IKK*β* to modulate phosphorylation of NF‐*κ*B in NPCs. Co‐IP results showed that Flag‐tagged STING clearly pulled down endogenous IKK*β* in both NPCs and N2A cells (Figure S7G,H, Supporting Information). To validate whether NF‐*κ*B activates ALX4 transcription, we first performed IUE experiment. ALX4 can partly rescue the abnormal distribution of GFP‐positive cells caused by NF‐*κ*B shRNA (Figure 4G). Results of chromatin immunoprecipitation (ChIP) assay showed that NF‐*κ*B mainly bound to the ALX4 promoter 3 kb upstream of the transcriptional start site (Figure 4H). To show that the regulatory effect of STING on the ALX4 promoter is via interaction with NF‐*κ*B, the binding enrichment of NF‐*κ*B on the ALX4 promoter region was detected by ChIP assay when STING signaling was suppressed or promoted. We found that STING was knockdown, the binding enrichment of NF‐*κ*B on the ALX4 promoter was reduced (Figure 4I). In contrast, the binding of NF‐*κ*B on ALX4 promoter was increased when STING signaling was promoted by cGAMP (Figure 4J). These results indicate that STING plays essential roles in promoting NF‐*κ*B binding onto the ALX4 promoter.

**Figure 5:**
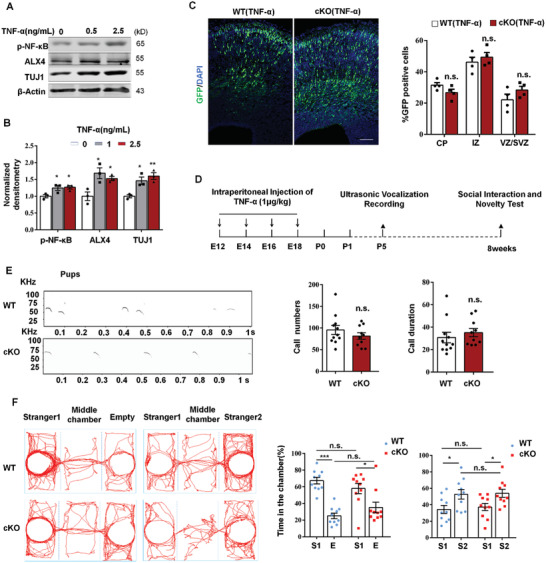
Rescue of impaired brain development and behaviors by low‐dose TNF‐*α* treatment. A) NPCs were treated with TNF‐*α* of indicated concentration for 2 d in proliferation medium. p‐NF‐*κ*B (p65), ALX4, and TUJ1 protein levels were analyzed by Western blot. B) Bar graph shows normalized densitometry of p‐NF‐*κ*B (p65), ALX4, and TUJ1 (*n* = 3 independent experiments). C) TNF‐*α* could partially rescue the abnormal GFP cell positioning in STING ablated embryonic cerebral cortex. For TNF‐*α* treatment, pregnant mice were intraperitoneally injected with TNF‐*α* (1 µg kg^−1^) at E12 and E14. IUE was performed at E13, and brains were collected and analyzed at E16. The percentage of GFP‐positive cells in the CP, IZ, and VZ/SVZ are shown in graph (*n* = 3 brains for all samples). D) Pregnant mice were intraperitoneally injected with TNF‐*α* (1 µg kg^−1^) at E12, E14, E16, and E18. And then, STING^fl/fl^ mice and STING^cKO^ mice were subjected to ultrasonic vocalization recording at P5 and three‐chamber social interaction test at eight weeks as indicated. E) Ultrasonic vocalization recording at P5 after TNF‐*α* treatment (*n* = 11 pups). F) Social interaction and novelty test of eight weeks mice after TNF‐*α* treatment (*n* = 10 mice). Error bars represent means ± SEM; *P* values were calculated by two‐tailed unpaired *t*‐test (B, C) or one‐way ANOVA with Tukey's post hoc (E, F), n.s., not significant, *P < 0.05, **P < 0.01, or ***P < 0.001.

TNF‐*α* can activate NF‐*κ*B through promoting phosphorylation of NF‐*κ*B.^[^ ^36^, ^37^ ^]^ In vitro experiment showed that protein levels of p‐NF‐*κ*B, Alx4, and primary neuronal marker TUJ1 were increased in NPCs after treatment with TNF‐*α* (**Figure** **5**A,B). We reasoned that abnormal embryonic neurogenesis and autism‐related phenotype in STING^cKO^ mice could be rescued by TNF‐*α*. To test this idea, we first performed IUE in pregnant mice, which were intraperitoneally injected with TNF‐*α* (1 µg kg^−1^). We found that TNF‐*α* could partially rescue the GFP^+^ cell positioning in STING ablated mice (Figure 5C). To test the effects of TNF‐*α* on autism‐related behaviors of STING^cKO^ mice, pregnant mice were intraperitoneally injected with TNF‐*α* as indicated (Figure 5D). Ultrasonic vocalization recording showed that there was no significant difference for call numbers or call duration between STING^cKO^ and wild‐type pups after the treatment of TNF‐*α* (Figure 5E). In the three‐chamber test, TNF‐*α* treatment completely rescued impaired social behaviors of the STING^cKO^ mice (Figure 5F).

### 2.6 Distinction of Cerebral Cortex Cell Types Is Observed in STING^cKO^ Mouse

**Figure 6:**
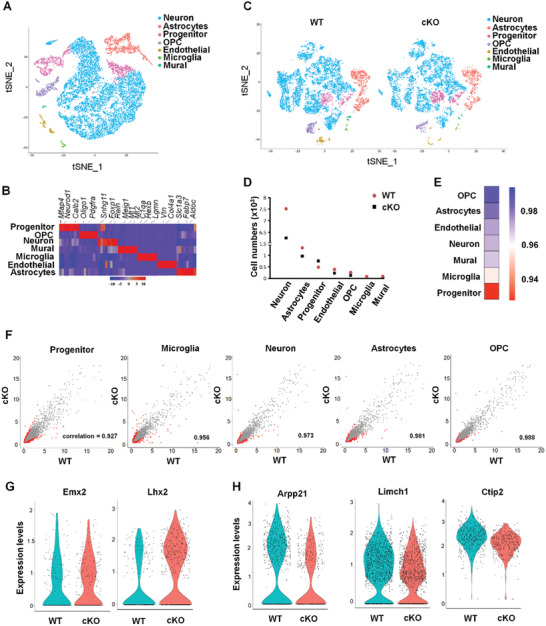
Distinction in single cell level is observed between neonatal WT and STING^cKO^ brain. A) tSNE plots of scRNA‐seq show unsupervised clusters of cells in brain cortex of P0. Seven major clusters; OPC, oligodendrocyte progenitor cell. B) Heatmap of each cluster's expression of the top eight markable genes per cluster. C) tSNE plots of scRNA‐seq show unsupervised clustering of cells in brain cortex of P0 WT and cKO mice. D) Cell numbers in different cluster between WT and cKO. E) Correlation analysis of each cluster from WT and cKO. Higher value (blue) indicates the gene expression pattern in that cluster is similar between WT and cKO samples. Lower value (red) indicates the gene expression pattern in that cluster is dissimilar between WT and cKO samples. F) Scatterplot analysis comparing average gene expression of genes in progenitor, microglia, neuron, astrocytes, and OPC between WT and cKO groups. Significantly differentially regulated genes (Log2 fold change ± 1) are displayed in red. G) Violin plots show expression distribution of progenitor cell gene Emx2 and Lhx2 in progenitor cluster, or H) neuron cell genes Arpp21, Limch1, and Ctip2 in neuron cluster of WT and cKO samples.

To clarify the abnormality in the STING^cKO^ cerebral cortex, we performed single‐cell RNA sequencing (scRNA‐seq) by using the 10× Genomics platform. ≈10 000 cells in both STING^fl/fl^ and STING^cKO^ samples passed quality control, and revealed seven clusters, which were displayed with t‐distributed stochastic neighbor embedding (tSNE) (**Figure** **6**A). We defined each group with multiple top specific genes and highlighted three genes for each cluster (Figure 6B). ScRNA‐seq analysis revealed that numbers of neurons were obviously decreased in STING^cKO^ mice and progenitor cells in STING^cKO^ mice were less than wild type (Figure 6C,D). In order to detect which cell types were the most affected in STING^cKO^, we analyzed the correlation of relative gene expression levels between WT and cKO for each cluster in an unbiased manner. Progenitor cells exhibited the lowest correlation values, indicating that progenitor cells have the largest variations in gene expression between WT and cKO compared with all other clusters (Figure 6E,F). We also found that the expression of Emx2 add Lhx2, two progenitor markers, was obviously dysregulated in progenitor cluster (Figure 6G). While for neuron‐specific markers in neuron group, STING^cKO^ displayed reduction in both number of expressing cells and average expression (Figure 6H).

### 2.7 Lack of STING in Human NPCs Exhibits Arrested Neuronal Differentiation

**Figure 7:**
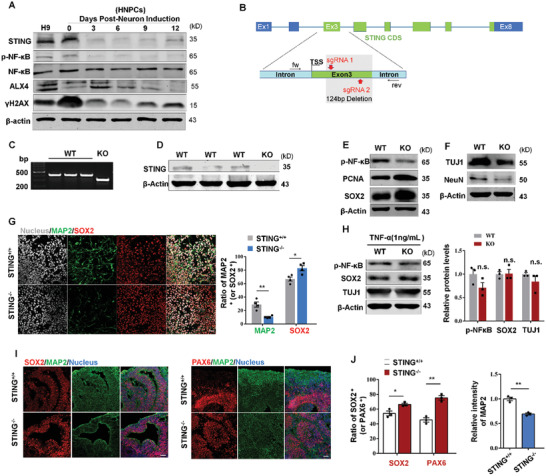
STING influences human NPCs differentiation. A) Human neural progenitor cells (HNPCs) were derived from H9 human embryonic stem cell (HESC) lines. Western blot for STING, p‐NF‐*κ*B, NF‐*κ*B, ALX4, and *γ*H2AX in H9 HESC, human NPCs, or differentiation cells at 0, 3, 6, 9, and 12 d postneuron induction. B) Schematic diagram for the generation of STING knockout HESC lines using CRISPR/Cas9. C)The KO allele was confirmed by genotyping PCR using primers indicated in (B). D) Western blots of the STING protein in the sgRNA‐treated WT and STING‐KO H9 HESC lines. E) Western blots showing p‐NF‐*κ*B, PCNA, and SOX2 level in human NPCs. F) Human NPCs were cultured in neuron differentiation medium for two weeks. Western blots showing TUJ1 and NeuN levels from WT and STING‐KO cell lines. G) WT and STING‐KO human NPCs were cultured in neuron differentiation medium for one week. Then cells were fixed and costained with anti‐SOX2 and anti‐MAP2 antibodies. Bar graph shows the ratio of MAP2 or SOX2‐positive cells in STING^+/+^ and STING^−/−^ groups (*n* = 4 independent experiments). H) STING^+/+^ and STING^−/−^ human NPCs were cultured in neuron differentiation medium containing 1 ng mL^−1^ TNF‐*α* for one week. Protein expression of p‐NF‐*κ*B, SOX2, and TUJ1 was analyzed by Western blot and STING‐KO cell lines (*n* = 3 independent experiments). I) Representative immunofluorescence images of hESC‐derived cortical organoids (35 d) from STING^+/+^ and STING^−/−^ groups for SOX2, PAX6, and MAP2. Scale bar, 50 µm. J) Quantification of the ratio of SOX2 or PAX6‐positive cells and relative intensity of MAP2 in organoids from STING^+/+^ and STING^−/−^ groups (*n* = 3 organoids). Error bars represent means ± SEM; two‐tailed unpaired *t*‐test, n.s., not significant, *P < 0.05, **P < 0.01, or ***P < 0.001.

To further probe the function of STING signaling in the brain and its conservation between mouse and human, we first detected protein levels of STING, p‐NF‐*κ*B, ALX4, and *γ*H2AX in H9 human embryonic stem cells (HESCs), human NPCs, and differentiated cells at 3, 6, 9, and 12 d postneuron induction. We found that STING, p‐NF‐*κ*B, and *γ*H2AX were highly expressed in HESCs and human NPCs, especially in HNPCs (**Figure** **7**A). Then, we generated STING KO H9 HESCs lines by CRISPR/Cas9‐mediated gene‐editing procedure (Figure 7B–D), and then differentiated them into NPCs. STING‐KO NPCs displayed reduced p‐NF‐*κ*B and increased PCNA and SOX2, two proliferation markers (Figure 7E). After two weeks of neuronal induction, TUJ1 and NeuN, two neuronal markers expressed at lower levels in STING‐KO cells than that in wild‐type cells (Figure 7F). Furthermore, immunostaining for SOX2 and mature neuronal marker MAP2 were consistent with observations from the Western blot, after one week of neuronal induction (Figure 7G). Next, we examined the role of TNF‐*α* in STING‐KO NPCs differentiation. Western blot analysis showed that there were no significant differences between STING‐KO and wild type in the p‐NF‐*κ*B, SOX2, and TUJ1 expression levels, after one week of neuronal induction (Figure 7H). To better understand the human cerebral cortex development, we generated human cerebral organoids using STING‐KO and wild‐type HESCs. We found that both STING‐KO and WT HESCs could successfully form cerebral organoids. However, immunostaining results showed that STING‐KO group displayed more signals of SOX2 and PAX6, and lower signal of MAP2 (Figure 7I,J). In summary, human STING‐KO NPCs showed delayed neuronal differentiation.

## 3 Discussion

Cortical neurogenesis is a sophisticated process that is strictly regulated by a large number of signaling molecules. Anything wrong in this process can cause abnormal brain functions.^[^ ^38^ ^]^ Our studies show that STING plays essential roles in cerebral cortex development. Unlike the well‐defined function of STING in the IFN response, STING deficiency in the embryonic brain promotes NPCs proliferation and inhibits neuronal differentiation. Increasing evidences documented that cGAS, a well‐defined upstream molecule of STING, promotes mitotic cell death by regulating the cGAS‐cGAMP‐STING‐IRF3 axis^[^ ^39^ ^]^ or inhibiting DNA repair by homologous recombination (HR)^[^ ^40^ ^]^ and also contributes to cellular senescence.^[^ ^41^ ^]^ Thus, cGAS likely acts as a negative regulator of cell proliferation. In the IUE experiment, we also found that cGAS could affect the normal neural development. Although most signaling pathways of STING depend on cGAS, cGAS‐independent STING pathways have been described.^[^ ^42^, ^43^ ^]^ Thus, the direct and indirect mechanisms between cGAS and STING in NPCs are also required.

In this study, we first confirmed that *γ*H2AX was highly expressed in early embryonic brain development, but further studies are necessary to verify the DNA damages in normal embryonic brain development. DNA damage induces the expression of STING^[^ ^11^ ^]^ and our work has also demonstrated that STING expression was correlated with *γ*H2AX as the proliferation of NPCs. In addition, ablation of cGAS can reduce STING expression, whereas knockdown of NF‐*κ*B does not affect STING transcriptional level. These results suggest that STING expression was mediated via DNA damage‐cGAS‐STING^[^ ^11^ ^]^ instead of the ATM‐TRAF6‐NEMO‐NF‐*κ*B axis^[^ ^44^ ^]^ during neuronal differentiation. We propose that STING probably plays a neuroprotective role in NPCs when there is a mass of DNA damage, but STING knockout does not cause cell death in cerebral cortex. Our findings also raise the question of whether there are other DNA damage‐signaling pathways influencing neuronal development.

We examined downregulated genes in STING^cKO^ mice via RNA‐sequencing analysis of E13 cerebral cortex. We find that the expression of ALX4 is reduced in STING^cKO^ relative to that in WT. The ALX4, aristaless‐like homebox‐4, is a paired‐like homedomain transcription factor.^[^ ^45^ ^]^ Previous study has found that ALX4 is a tumor suppressor in hepatocellular carcinoma progression.^[^ ^46^ ^]^ However, less is known about the effect of ALX4 in the development of the brain. In this work, we show that the depletion of ALX4 promotes the proliferation and inhibits the differentiation of NPCs. Furthermore, increasing ALX4 levels can partially rescue the abnormal neurogenesis resulting from STING knockdown. Our findings indicate that ALX4 is a key effector in brain development. Nevertheless, discovering more details of ALX4 in brain is remained to be resolved.

Our studies also provide evidences that STING promotes the phosphorylation of NF‐*κ*B. It is known to be a key regulator of multiple aspects of cellular events. But few studies focus on the function of NF‐*κ*B in embryonic development. Herein, we report that NF‐*κ*B can bind to the promoter of ALX4 in NPCs, and this process is controlled by STING. Furthermore, our study demonstrates that STING is important for dendritic morphology. Defect of dendrite morphogenesis results in neuronal dysfunctions, which are associated with neurodevelopmental disorders, such as depression and autism.^[^ ^3^, ^47^ ^]^ STING^cKO^ mice display autistic‐like behaviors. Although STING has never been studied in autism, previous research has found that ALX4 is a risk gene for autism.^[^ ^48^ ^]^ Interestingly, we find that low‐dose TNF‐*α*, an activator of NF‐*κ*B, can rescue some autistic‐like behaviors in STING^cKO^ mice. Although we have identified that low‐dose TNF‐*α* can increase the phosphorylation of NF‐*κ*B and the expression of ALX4, a better understanding of immune responses among mice model of neurodevelopmental disorders is likely to discover more potential drugs. We identified that both IRF3 and TBK1 were also phosphorylated in embryonic cerebral cortex, however only p‐NF‐*κ*B was downregulated in STING^cKO^. It means that STING undergoes a different mode of activation during brain development. Although the molecular detail of STING signaling pathway is best characterized for eliciting innate immune response, more evidence indicates that STING can also promote ER stress,^[^ ^49^ ^]^ apoptosis,^[^ ^50^ ^]^ inflammasome activation,^[^ ^51^ ^]^ and autophagy.^[^ ^52^ ^]^ In our study, STING influences the NPCs differentiation during embryonic brain development. Thus, this finding expands the biological function of STING.

## 4 Experimental Section

##### Animals

C57BL/6 mice (8–10 weeks of age) were obtained from the Vital River. STING floxed mice were obtained from the Shanghai Model Organisms Center (Shanghai, China). To generate STING^fl/fl^ mice, STING floxed mice were crossed with C57BL/6J mice, and then the F1 offsprings were bred to obtain STING^fl/fl^ mice. STING^cKO^ mice were generated by crossing Nestin‐Cre transgenic mice (obtained from Jackson Laboratories) with STING^fl/fl^ mice. All animal experiments were performed under the ethical guidelines of the Animal Care and Use Committee of Institute of Zoology, Chinese Academy of Sciences.

##### Cell Culture

HEK293T and mouse neuroblastoma N2a cells were cultured in DMEM (Life Technologies) supplemented with 10% fetal bovine serum (BIOCHROME) and 1% penicillin/streptomycin. H9 human ES cells were cultured in Essential 8 medium (Thermo, A1517001) and maintained on Matrigel‐coated six‐well plates (Corning, 354277). Primary neural progenitor cells were isolated from E13 cerebral cortices and seeded onto six‐well or 24‐well plates coated with 10 µg mL^−1^ poly‐d‐lysine (Sigma‐Aldrich) and 10 µg mL^−1^ laminin (Invitrogen). Primary NPCs were cultured in 50% DMEM/F12 (Invitrogen) and 50% neurobasal‐A medium (Invitrogen) supplemented with 1% penicillin‐streptomycin (Invitrogen), 1× GlutaMAX (Invitrogen), 1× nonessential amino acids (Invitrogen), 2% B27 supplement (without VA) (Invitrogen), 5 ng mL^−1^ basic fibroblast growth factor (Invitrogen), and 2.5 ng mL^−1^ epidermal growth factor (Invitrogen). The differentiation medium was consisted of low‐glucose DMEM (Gibco) with 1% penicillin‐streptomycin and 2% B27 supplement (Invitrogen). Intracellular delivery of CMA and cGAMP was performed using Lipofectamine MessengerMAX. The other reagents are listed in Table S1 (Supporting Information).

##### Generation of STING‐KO hPSC Lines

H9 cells were dissociated into single cell by Accutase (Thermo, A1110501), when cells grew to 70% confluence. Then the cells were washed by DPBS two times and half a million cells were resuspended in P3 solution (Lonza V4XP‐3012) containing 1.5 µg lentiCRISPR‐STING‐sgRNA1 and 1.5 µg lentiCRISPR‐STING‐sgRNA2. Nucleofection was performed using LONZA 4D‐Nucleofector by program CB‐150. After nucleofection, H9 cells were seeded onto six‐well plates coated with Matrigel and cultured in E8 medium containing 5 × 10^−6^ m Y27632 (Sigma) for the first 24 h. Puromycin (0.5 µg mL^−1^) was added from day 3 to 9. About two weeks later, H9 colonies were picked and plated onto Matrigel coated 24‐well plates. After PCR genotyping and Western blot analysis, positive colonies (STING‐KO) were expanded and cryopreserved. Nucleofected cells having STING (STING‐WT) served as controls.

##### Lentivirus Production and Infection

Lentiviral package plasmids (Addgene) and the core plasmids (pSicoR, PCDH) were transfected into 293T cells by GenEscort II (Wisegen). The supernatant medium with lentivirus was collected at 48 h after transfection and centrifuged at 3000 rpm for 5 min to remove the cell debris. For lentivirus infection, half primary NPCs medium was replaced with virus suspension and 2 µg mL^−1^ polybrene. 8 h later, the supernatant medium was changed with new differentiation or proliferation medium. The plasmids are listed in Table S1 (Supporting Information).

##### Western Blotting and Immunoprecipitation

For Western blotting, cell pellets or tissues were lysed in RIPA lysis buffer (Solarbio) containing 1% PMSF and 1% Cocktail (Sigma). Cell debris was removed by using microcentrifuge for 15 min, 12 000 rpm, 4 °C. Then the protein samples were boiled in 4× loading buffer and loaded onto 12% sodium lauryl sulfate polyacrylamide gel electrophoresis (SDS‐PAGE) gels. Next, proteins were transferred to nitrocellulose membrane and blocked by 5% nonfat milk (PBS+0.05% Tween20) at room temperature for 1 h. The protein bands were analyzed via immunoblotting. For immunoprecipitation, lysing cells in lysis buffer (NP‐40 0.5% (v/v), NaCl 300 × 10^−3^ m, EDTA 1 × 10^−3^ m, Tris‐HCl 20 × 10^−3^ m (pH 8.0), 1% PMSF, 1% cocktail), taking 600 µg proteins to suspend with Dynabeads Protein A (Invitrogen) or anti‐DDDDK‐tag magnetic beads (MBL), overnight at 4 °C. Using 600 µl cold 0.02% PBST to wash the beads three times softly and the bound proteins were analyzed by Western blot. The antibodies are listed in Table S1 (Supporting Information).

##### Immunofluorescence

Mouse brain or organoids slices or cultured cells were washed with washing buffer (1% Triton X‐100 in 1× PBS), fixed in 4% paraformaldehyde in 1× PBS for 20 min, blocked by 5% BSA (in washing buffer) for 45 min. Sections or cells were incubated with primary antibodies in 1% BSA overnight at 4 °C. After washing, fluorescence‐labeled secondary antibodies in 1%BSA were added to incubate samples for 30 min. After washing, samples were counterstained with 4′,6‐diamidino‐2‐phenylindole (DAPI) for 3 min. Images were acquired using Carl Zeiss LSM780 confocal microscope. For cytosolic DNA staining, cells were treated with 0.02% saponin (Sigma) for 5 min after fixation to permeate plasma membrane selectively. 5% BSA in PBS was used as blocking solution during antibody staining. The antibodies are listed in Table S1 (Supporting Information).

##### In Utero Electroporation

The detailed protocols were performed previously.^[^ ^53^ ^]^ Briefly, pregnant mice were deeply anesthetized by pentobarbital sodium (70 µg g^−1^), and plasmid DNA with 0.2% Fast Green (Sigma) were injected into the fetal lateral ventricle through a glass micropipette. After electroporation, the electroporated mice were sacrificed for phenotype analysis at indicated days. The plasmids are listed in Table S1 (Supporting Information).

##### Real‐Time PCR Analysis

Total RNA was extracted using TRIzol (Invitrogen, 15596) method. The first‐strand cDNA was obtained by the FastQuant RT Kit (TIANGEN). Quantitative RT‐PCR was conducted in 20 µL reaction mixture using the SYBR Green PCR Kit (TIANGEN) with the 7500 real‐time PCR system (Applied Biosystems). The primer sequences are listed in Table S2 (Supporting Information).

##### RNA‐Sequencing Analysis

Total RNA of E13 cerebral cortices of STING cKO and WT mice was extracted by TRIzol method. Then, total RNA was quantified and quality controlled by Agilent 2100 Bioanalyzer (Annoroad Genomics). After cDNA library construction, high‐throughput sequencing was performed through Illumina HiSeq 2500 platform (Annoroad Genomics). Differential expressed genes were identified between WT and cKO. Gene ontology analysis was also performed. Genes with ≥2‐fold change and *P* value ≤ 0.05 were considered as statistically significant. The data are accessible in NCBI's GEO with accession number: GSE146455.

##### Human NPC Induction

Human NPC induction was based on previous study.^[^ ^54^ ^]^ In brief, when H9 embryonic stem cells reached 70–80% in six‐well plate, medium was replaced with neural progenitor cell induction medium including 50% (v/V) DMEM/F12 (Invitrogen), 50% (v/V) neurobasal medium (Invitrogen), 1× B27 supplement (Invitrogen), 1× N2 supplement (Invitrogen), 1× GlutaMAX, 2 × 10^−6^ m dorsomorphin (Sigma), 10 ng mL^−1^ hLIF (Millipore), 4 × 10^−6^ m SB431542 (Sigma), 4 × 10^−6^ m CHIR99021 (Sigma), and 0.2 × 10^−6^ m compound E (EMD Chemicals). Cells were cultured for 7 d and changed the medium every other day. Then, cells were separated into single cell by Accutase and transferred to six‐well (100 000/well) or 24‐well (8000/well) matrigel‐coated plates, and maintained in human neural progenitor medium (50% (v/v) neurobasal medium, 50% (v/v) DMEM/F12, 1× B27, 1× N2 supplement, 1× GlutaMAX, 10 ng mL^−1^ hLIF, 3 × 10^−6^ m CHIR99021, and 3 × 10^−6^ m SB431542). For neuronal differentiation, human NPCs were cultured for 3 d and then cells were incubated in neuronal induction medium (DMEM/F12 medium containing 1× B27 supplement, 1× N2 supplement, 400 × 10^−6^ m dbcAMP (Sigma), 10 ng mL^−1^ BDNF (Peprotech), and 10 ng mL^−1^ GDNF (Peprotech)). Laminin (Sigma, 1 µg mL^−1^) was added after 2 d culture. Cells were cultured for 7 or 14 d and changed the medium every other day.

##### ChIP Analysis

ChIP analysis was conducted as follows. Cultured cells were maintained in 1% formaldehyde solution (50 × 10^−3^ m, HEPES‐KOH, 100 × 10^−3^ m NaCl, pH 7.5, 0.5 × 10^−3^ m EGTA 1 × 10^−3^ m EDTA, and 1% formaldehyde) at room temperature for 15 min, then added 2.5 m glycine to terminate the reaction. After rinsing three times with cold PBS, the cells were collected in lysis buffer 1 (140 × 10^−3^ m NaCl, 50 × 10^−3^ m HEPES‐KOH (pH 7.5), 1 × 10^−3^ m EDTA, 10% glycerol, 0.25% Triton X‐100, 0.5% NP‐40, and 1× PMSF) for 15 min. Next, the lysates were isolated at 5000 rpm for 10 min at 4 °C and resuspended in lysis buffer 2 (200 × 10^−3^ m NaCl, 10 × 10^−3^ m Tris‐HCl (pH 8.0), 0.5 × 10^−3^ m EGTA, 1 × 10^−3^ m EDTA, 1× PMSF). And then rocked at room temperature gently for 10 min. Samples were sonicated (Scientz‐IID) in lysis buffer 3 (50 × 10^−3^ m Tris‐HCl (pH 8), 1% SDS, 10 × 10^−3^ m EDTA, 1× PMSF). The samples were incubated with 50 µL of Dynabeads Protein A or anti‐HA‐tag beads overnight at 4 °C. Beads were washed three times with low‐salt (1% Triton X‐100, 0.1% SDS, 2 × 10^−3^ m EDTA, 150 × 10^−3^ m NaCl, Tris‐HCl (pH 8)) and high‐salt buffer (1% Triton X‐100, 0.1% SDS, 2 × 10^−3^ m EDTA, 500 × 10^−3^ m NaCl, Tris‐HCl (pH 8)) and then incubated overnight at 65 °C. Input or immunoprecipitated DNA was extracted by TIANamp Genomic DNA Kit. Quantities of DNA were performed by real‐time PCR. The primers are listed in Table S2 (Supporting Information).

##### Open Field Test

Individual mice (8–12 weeks old) were placed into an enclosed arena (40 cm × 40 cm × 40 cm). The movement duration in the center and total distance was recorded by the Topscan behavioral analysis software (Clever Sys Inc., Reston, VA) for 5 min.

##### Elevated‐Plus Maze

Testing was conducted as previously described.^[^ ^55^ ^]^ The maze has two closed arms (25 cm × 5 cm, 15 cm transparent walls) and two open arms (25 cm × 5 cm). The maze stood 50 cm above the floor. Mice were placed in the center square of the maze facing a closed arm and allowed to explore for 5 min.

##### Forced Swim Test

Testing was conducted as previously described.^[^ ^56^ ^]^ In brief, the forced swim test was performed in a 35 cm high and 20 cm diameter cylindrical glass container which was filled with 25 °C water. Behavior was recorded for 6 min, and the last 4 min were used to analyze.

##### Y‐Maze

Spontaneous alternation testing was performed in the Y‐maze as previously described.^[^ ^57^ ^]^ During the training phase, individual mice were allowed to explore start arm and old arm with the novel arm blocked for 10 min. After 30 min, individual mice were placed into start arm of maze and were allowed to explore the three arms for 5 min.

##### Three‐Chamber Test

The test was conducted as described previously.^[^ ^55^ ^]^ The three‐chamber test comprised of three parts. The first part is habituation, empty wire cages put in the right and left chambers, and the individual test mice were allowed to habituate the environment for 10 min. After habituation, a gender‐ and age‐matched unfamiliar mouse (Stranger 1) was placed in left wire cage. Then, the test mouse was placed in the middle chamber and allowed to explore the chamber for 10 min. In the third part, a second gender‐ and age‐matched unfamiliar mouse (Stranger 2) was placed in the right empty wire cage. The trajectory and time spent in each chamber of the test mice were analyzed by the Topscan behavioral analysis software.

##### Ultrasonic Vocalization

The test was conducted during 9:00–16:00 as reported previously.^[^ ^58^ ^]^ The pups of WT or STING^cKO^ at P5 were removed individually from their mother and littermates, and placed into a sound attenuating box for 5 min. USV emission was recorded using Avisoft Recorder software (Version 4.2) with Ultra Sound Gate Condenser Microphone CM16 (Avisoft Bioacoustics). The microphone was 10 cm high above the pups, and was sensitive to a flat frequency response (±6 dB) between 25 and 140 kHz. The analysis of recordings was performed using the Avisoft SASLab Pro (Version 5.20) as protocol. The fast Fourier transform was performed (512 FFT length, Hamming window, 100% frame, and 75% overlap of time window). The frequency and time resolution of spectrograms was 488 Hz and 0.512 ms, respectively. Call detection was recorded with hold time: 10 ms, amplitude threshold 40 dB, high‐pass filter 30 kHz, and noise reduction filter 40 dB. The entire session is 5 min. For each test, the sound‐attenuating box was cleaned with 70% ethanol and water.

##### Marble Burying Test

The marble test was conducted in familiar and standard rodent house cages. Cages contained 5 cm deep fresh bedding. 20 standard black glass marbles (diameter: 15 mm) were placed on the surface of the fresh bedding in four rows. The individual test mice were gently placed on the house cages and allowed to explore for 30 min in undisturbed environment. The buried marbles were counted, and a buried marble was scored if two‐thirds was covered by bedding.

##### Generation of Cerebral Organoids

Generation of cortical organoids from H9 hESC lines was conducted as previous study.^[^ ^59^ ^]^ Briefly, hESCs were dissociated to single cell using Accutase for 6 min at 37 °C. Cells were collected in E8 medium with ROCK inhibitor (50 × 10^−6^ m) and about 10 000 cells were plated in each well of low‐attachment 96 V plate (Sumitomo Bakelite) to form EBs. The next day, the medium was changed completely with low‐bFGF hESC medium (80% (v/v) DMEM‐F12, 8.5% (v/v) KOSR, 1.5% (v/v) ESC‐quality FBS, 1× GlutaMAX, 1× MEM‐NEAA, and 4 ng mL^−1^ bFGF). The EBs were fed every other day without disturbing the EB, for 5 d. Then, each EB was transferred to low‐attachment 24‐well plate containing neural induction medium (DMEM‐F12 with 1× N2 supplement, 1× GlutaMAX, 1× MEM‐NEAA, and 1 µg mL^−1^ heparin). After 5 d in neural induction medium, the aggregates were transferred one by one to each matrigel droplet and cultured in 10 mm dish with cerebral differentiation medium (50% (v/v) neurobasal medium, 50% (v/v) DMEM/F12, 1× B27, 1× N2 supplement, 1× GlutaMAX, and 0.02% (v/v) human insulin) without vitamin A. After 4 d in static culture, the embedded organoids were transferred to a 100 mL^−1^ spinning bioreactor and cultured in 70–85 mL cerebral differentiation medium containing vitamin A. The medium was changed completely every week.

##### Single‐Cell RNA Sequencing

Neonates brain was removed from skull on a frozen surface. The cerebral cortex was collected and dissociated using papain (20 U mg^−1^; Worthington) diluted in artificial cerebrospinal fluid (aCSF, 87.0 × 10^−3^ m NaCl, 26.0 × 10^−3^ m NaHCO3, 2.5 × 10^−3^ m KCl, 75.0 × 10^−3^ m sucrose, 20.0 × 10^−3^ m glucose, 1.0 × 10^−3^ m CaCl_2_, 7.0 × 10^−3^ m MgSO_4_, 1.25 × 10^−3^ m NaH_2_PO_4_ at a pH of 7.4 and equilibrated in 5% CO_2_ and 95% O_2_) for 20 min at 37 °C. Then cell suspensions were filtered through a 40 µm cell strainer and 200 *g* for 5 min. Cells were washed three times with 10 mL ice‐cold aCSF at 200 *g* for 5 min. Cells were then resuspended in ice‐cold D‐PBS containing 1% BSA at a concentration of about 100 000 cells mL^−1^, and performed with 10× Genomics Chromium Single Cell Kit for Single‐Cell RNA‐sequencing. The data are accessible in NCBI's GEO with accession number: GSE146456.

##### Statistical Analysis

The statistical analyses were conducted using GraphPad Prism 6.0 or Excel 2016. All data are represented as mean ± SEM. Statistical comparisons were conducted using the unpaired *t*‐test or one‐way analysis of variance (ANOVA) with Tukey's post hoc tests (*P < 0.05, **P < 0.01, ***P < 0.001, or n.s., not significant) (Table S3, Supporting Information).

## Conflict of Interest

The authors declare no conflict of interest.

## Author Contributions

J.J. and D.Z. designed the research; D.Z. performed and analyzed the experiments, and wrote the manuscript. C.L. did some plasmids construction and in utero electroporation. H.L. provided some suggestions for the research. J.J. supervised the project and obtained funding support.

## Supporting information

Supporting Information
