# Supplementary material for: Deficiency of STING Signaling in Embryonic Cerebral Cortex Leads to Neurogenic Abnormalities and Autistic‐Like Behaviors
Source: Adv Sci (Weinh). 2020 Nov 3;7(23):2002117. doi: 10.1002/advs.202002117 (PMC7710002; doi:10.1002/advs.202002117)
Supplement: Supplementary file 1 — Supporting Information [file ADVS-7-2002117-s001.pdf]

© 2020 Wiley-VCH GmbH

ADVANCED SCIENCE
Open Access

Supporting Information

for *Adv. Sci.*, DOI: 10.1002/advs.202002117

Deficiency of STING-signaling in embryonic cerebral cortex leads to neurogenic abnormalities and autistic-like behaviors

*Dongming Zhang, Chang Liu, Hong Li, and Jianwei Jiao**

Supporting Information

Deficiency of STING-signaling in embryonic cerebral cortex leads to neurogenic abnormalities and autistic-like behaviors

*Dongming Zhang, Chang Liu, Hong Li, Jianwei Jiao**

Supplementary Table 1: Reagent or Resource

REAGENT or RESOURCE	SOURCE	IDENTIFIER
Antibodies		
rabbit anti-STING	Cell Signaling Technologies	Cat# 13647; RRID: AB_2732796
rabbit anti-γH2AX	Cell Signaling Technologies	Cat# 9718; RRID: AB_2118009
rabbit anti-SOX2	Cell Signaling Technologies	Cat# 3728; RRID: AB_2194037
mouse anti-MAP2	Millipore	Cat# MAB3418; RRID: AB_94856
mouse anti-dsDNA	Santa Cruz Biotechnology	Cat# sc-58749; RRID: AB_783088
rabbit anti-STING	Proteintech	Cat 19851-1-AP; RRID: AB_10665370
mouse anti-Nestin	Millipore	Cat# MAB353; RRID: AB_94911
rabbit anti-Pax6	Abcam	Cat# ab5790; RRID: AB_305110
rabbit anti-Pax6	Millipore	Cat# AB2237; RRID: AB_1587367
rabbit anti-Tbr2	Abcam	Cat# ab23345; RRID: AB_778267
rabbit anti-Tuj1	Sigma-Aldrich	Cat# T2200; RRID: AB_262133
mouse anti-Tuj1	Millipore	Cat# MAB1637; RRID: AB_2210524
mouse anti-β-actin	Proteintech	Cat# 60008-1-Ig; RRID: AB_2289225
rat anti-Ctip2	Abcam	Cat# ab18465; RRID: AB_2064130
mouse anti-SATB2	Abcam	Cat# ab51502; RRID: AB_882455
rat anti-BrdU	Abcam	Cat# ab6326; RRID: AB_305426
mouse anti-ALX4	Santa Cruz Biotechnology	Cat# sc-33643; RRID: AB_2226342
rabbit anti-Flag	Sigma-Aldrich	Cat# F1804; RRID: AB_262044
rabbit anti-IgG	Bioss	Cat# bs-0295p; RRID: AB_2811143
rabbit anti-p-NF-κB (p65) (Ser536)	Cell Signaling Technology	Cat# 3033; RRID: AB_331284
mouse anti-NF-κB p65	Cell Signaling Technology	Cat# 6956; RRID: AB_10828935
rabbit anti-p-STAT6 (Tyr641)	Cell Signaling Technology	Cat# 9361; RRID: AB_331595
rabbit anti-p-TBK1(Ser172)	Cell Signaling Technology	Cat# 5483; RRID: AB_10693472
rabbit anti-IKKβ	Bioss	Cat# bs-2910R; RRID: AB_10855626
rabbit anti-p-IRF3 (Ser396)	Bioss	Cat# bs-3195R; RRID: AB_10857145
rabbit anti-NeuN	Abcam	Cat# ab177487; RRID: AB_2532109
rabbit anti-PCNA	Santa Cruz Biotechnology	Cat# sc-56; RRID: AB_628110
goat anti-SOX2	R&D Systems	Cat# AF2018; RRID: AB_355110
Chemicals, Peptides, and Recombinant Proteins		
2’ 3’-cGAMP	Sigma-Aldrich	Cat# SML1229
CMA	Sigma-Aldrich	Cat# 17927
Ara-c	Sigma-Aldrich	Cat# C1768
Etoposide	Sigma-Aldrich	Cat# E1383
mouse TNF-α	Cell Signaling Technology	Cat# 5178SF

human TNF-α	Cell Signaling Technology	Cat# 8902S
Critical Commercial Assays		
P3 Primary Cell 4D-Nucleofector™ X Kit L	Lonza	Cat# V4XP-3024
In Situ Cell Death Detection Kit	Roche	Cat# 12156792910
SYBR Green PCR Kit	TIANGEN	Cat# FP205
Deposited Data		
Raw and analyzed data of bulk RNA-seq	This paper	GEO: GSE146455
Raw and analyzed data of single-cell RNA-seq	This paper	GEO: GSE146456
Experimental Models: Cell Lines		
HEK293 Cell Line	ATCC	ATCC CRL-1573; RRID: CVCL_0045
Neuro-2a	ATCC	ATCC CCL-131; RRID: CVCL_0470
H9 hESCs	WiCell	RRID: CVCL_9773
Experimental Models: Organisms/Strains		
C57BL/6	Jackson Laboratory	Stock No: 000664; RRID: IMSR_JAX:000664
Mouse: STING$^{fl/fl}$	Shanghai Model Organisms Center	N/A
Mouse: Nestin-cre	Jackson Laboratory	Stock No: 019103; RRID: IMSR_JAX:019103
Oligonucleotides		
Primers for QPCR, see Table S1	This paper	N/A
Recombinant DNA		
pSicoR-GFP	Addgene	N/A
pSicoR-GFP-STING-shRNA1: ATGATTCTACTATCGTCTTAT	This paper	N/A
pSicoR-GFP-STING-shRNA2: ATGATTCTACTATCGTCTTAT	This paper	N/A
pSicoR-GFP-cGAS-shRNA: GAGATTGAAACGCAAAGATAT	This paper	N/A
pSicoR-GFP-Alx4-shRNA: GAGTTGCCTCCTGACTCTGAA	This paper	N/A
pSicoR-GFP-Htr2c-shRNA: CGGCATACCAATGAACGTGTA	This paper	N/A
pSicoR-GFP-Slc4a5-shRNA: CTGAGATGCCAGCTATGCAC	This paper	N/A
pSicoR-GFP-Dnah6-shRNA: CTTACGCGCTGCGGGATTTAT	This paper	N/A
pSicoR-GFP-NF-κB (p65)-shRNA: GATCAATGGCTACACAGGA	This paper	N/A
I pSicoR-GFP-KKβ-shRNA: GATGAGTCTCCTCCGGAATAA	This paper	N/A
pSicoR-GFP-IRF3-shRNA: GGTTGTTCCTACATGTCTTAA	This paper	N/A
pSicoR-GFP-TBK1-shRNA: GTTTAAAGATAAGTCGGAA	This paper	N/A
pSicoR-GFP-STAT6-shRNA: CCGGGATCTTGCTCAGTTAAA	This paper	N/A
PCDH	System Biosciences	Cat# CD511B-1
PCDH-3flag-STING	This paper	N/A
PCDH-3flag-cGAS	This paper	N/A

PCDH-3HA-ALX4	This paper	N/A
PCDH-3HA- NF-κB (p65)	This paper	N/A
lentiCRISPR V2	Addgene	Cat# 52961
lentiCRISPR V2-sgRNA (STING)1: TCCCGTGTCCCAGGGGTCAC	This paper	N/A
lentiCRISPR V2-sgRNA (STING)2	This paper	N/A
Software and Algorithms		
ImageJ	Schneider et al., 2012	https://imagej.nih.gov/ij/
Zen Microscope software	Zeiss	https://www.zeiss.com/microscopy/int/products/microscope-software/zen.html
ABI7500 real-time PCR system	Applied Biosystems	Applied Biosystems
Odyssey imaging system	LI-COR Biosciences	LI-COR Biosciences
Avisoft RECORDER USGH	Avisoft Bioacoustics	https://www.avisoft.com/downloads/
Avisoft SASLab Pro	Avisoft Bioacoustics	https://www.avisoft.com/downloads/
Other		
DAPI	ThermoFisher	Cat# D3571
Lipofectamine™ MessengerMAX™ Transfection Reagent	ThermoFisher	Cat# LMRNA001
anti-HA-tag magnetic beads	MBL	Cat# M132-11
anti-flag-tag magnetic beads	MBL	Cat# M185-11
Protein A Magnetic Beads	Invitrogen	Cat# 10002D

Supplementary Table 2: QPCR Primers

qPCR oligonucleotides	Forward sequence (5' > 3')	Reverse sequence (5' > 3')
mAscl1	GCAACCGGGTCAAGTTGGT	CAAGTCGTTGGAGTAGTTGGG
mBrn2	GCAGCGTCTAACCACTACAGC	GCGGTGATCCACTGGTGAG
mNeuroD1	ATGACCAAATCATACAGCGAGAG	TCTGCCTCGTGTTCCTCGT
mMyt1L	GGGAGAAGGGATAACCTGAGAT	CGTGGCCGTTTCCATCACA
mNkx2.1	CCAAGCTTAGGCATGGCACA	GGGTTATGCTGAAGACTTTCCT
mP21	GTCCAATCCTGGTGATGTCCG	AAGTCAAAGTTCCACCGTTCTC
mTuj1	CCAGTGCGGCAACCAGATAGG	AAAGGCGCCAGACCGAACACT
mβ-actin	CGCCACCAGTTCGCCATGGA	TACAGCCCGGGGAGCATCGT
mIL-6	TAGTCCTTCCTACCCCAATTTCC	TTGGTCCTTAGCCACTCCTTC
mIFNβ	CAGCTCCAAGAAAGGACGAAC	GGCAGTGTAACTCTTCTGCAT
mTNF-α	TGGAACTGGCAGAAGAG	CCATAGAACTGATGAGAGG
mIFIT2	GCTCTGGAAAAGGACCCGAA	GCTTCAGTGCCAAGAGGACT
mIL-1β	CACCTTCTTTTCCTTCATCTTTG	GTCGTTGCTTGGTTCTCCTTGTA
mIL-12B	TGGTTTGCCATCGTTTTGCTG	ACAGGTGAGGTTCACTGTTTCT
mCCL2	TTAAAAACCTGGATCGGAACCAA	GCATTAGCTTCAGATTTACGGGT
mSTING	AAATAACTGCCGCCTCATTG	TGGGAGAGGCTGATCCATAC
mcGAS	ACCGGACAAGCTAAAGAAGGTGCT	GCAGCAGGCGTTCCACAACTTTAT
mHtr2c	AATAGGGGGCAACATTCTCG	GGGGCACAAATATCTAGGTAAAG
mSlc4a5	CAAACATCAGAATTGAGCCACC	CCAGACACAGTCCTCCAAAGAA
mDnah6	CGATGGTCCGTTTGATTTAC	CTGGCAGTGTTTACTGTTAGG
mTBK1	GACATGCCTCTCTCCTGTAGTC	GGTGAAGCACATCACTGGTCTC
mIRF3	CGGAAAGAAGTGTTGCGGTTAGC	CAGGCTGCTTTTGCCATTGGTG
mNF-κB(p65)	TACCCTGTACACCTGGCAGT	AAGGTCCGGGCATTCACATT
mIkkβ	CTGTGCACGTCATTTGTGGG	CTTCTGCCGGACTTTGGAGT
mAlx4	TGCTACGCCAAAGAGAGCAA	AGTGAAGGTGGTTCGGTTCC
ALX4-5K	ACTCAGTGACGGACAGACGG	GAAGGTAGGCGAGGCAGAAA
ALX4-3K	TTTCCCATTGGGCAGTCAAG	TCCCTCTCCCTCCCTCTCTC
ALX4-1.5k	TTCTGGCACAATAGACATTCA	ATCCCACATCTGGTTTTCAT
ALX4-300bp	AACCAAAAGCTCCGTAAAAA	GTCAGGGGGGAAAAAATCAC

Supplementary Table 3. Statistics and Quantification

Main figures

Figure	Number of subjects	Test	Key comparisons
1A	All conditions = 5	One-way ANOVA	F (2, 12) = 101.8 P < 0.0001
1D	All conditions = 6	Unpaired Two-Tailed t-test	STING: t = 4.444, df =10, p = 0.0012<br>γH2AX: t=7.240, df=10, P<0.0001
1F	All conditions = 5	Unpaired Two-Tailed t-test	VZ/SVZ<br>Sh1: p = 0.033<br>Sh2: p = 0.014<br>IZ<br>Sh1: p = 0.25<br>Sh2: p = 0.21<br>CP<br>Sh1: p = 0.0003<br>Sh2: p < 0.0001
1G	All conditions = 3	Unpaired Two-Tailed t-test	il12b: p = 0.8667782<br>ifnb: p = 0.09200238<br>ifit2: p = 0.14647119<br>il1b: p = 0.80449185<br>il6: p = 0.01532683

			tnfa: p = 0.47430475<br>ccl2: p = 0.080310696
1H	All conditions = 4	Unpaired Two-Tailed t-test	Brn2: p = 0.028<br>Myt1l: p = 0.0007<br>NKX2.1: p < 0.0001<br>p21: p < 0.0001<br>Neurod1: p =0.00027<br>Tuj1: p = 0.003<br>Ascl1: p = 0.014
1I	All conditions = 4	Unpaired Two-Tailed t-test	MAP2: t = 4.196, df = 6, p = 0.0057<br>SOX2: t=2.474, df=6, p = 0.0482
2B	All conditions = 4	Unpaired Two-Tailed t-test	CP<br>wt(control) vs cko (control): p = 0.000720678<br>cko(control) vs cko (sting): p = 0.024<br>IZ<br>wt(control) vs cko (control): p = 0.389382375<br>cko(control) vs cko (sting): p = 0.863948892<br>VZ/SVZ<br>wt(control) vs cko (control): p = 0.043135388<br>cko(control) vs cko (sting): p = 0.048898874
2C	All conditions = 3	Unpaired Two-Tailed t-test	TBR2: t = 2.79, df = 4, p = 0.049<br>SOX2: t=4.454, df=4, p = 0.0112<br>PAX6: t=2.474, df=4, p = 0.039
2D	All conditions = 3	Unpaired Two-Tailed t-test	t=5.939, df=4, p= 0.0040
2E	All conditions = 4	Unpaired Two-Tailed t-test	Ctip2: t=4.735, df=4, p = 0.0091<br>SATB2: t=5.013, df=4, p= 0.0074
2F	All conditions = 3	Unpaired Two-Tailed t-test	t=3.336, df=4, p=0.0289
2H	All conditions = 6	Unpaired Two-Tailed t-test	t=3.882, df=10, p= 0.0031
3B	All conditions = 12	One-way ANOVA	F = 5.3601, p = 0.0295
3C	All conditions = 12	One-way ANOVA	F = 0.0112, p= 0.9167
3E	All conditions = 12	One-way ANOVA	F = 5.7453, p= 0.0255
3F	All conditions = 12	One-way ANOVA	F = 2.2745, p= 0.1457
3I	All conditions = 12	One-way ANOVA	WT: F = 74.1511, p < 0.0001<br>cKO: F = 1.8954, p= 0.1824
3J	All conditions = 12	One-way ANOVA	WT: F = 28.6384, p < 0.0001<br>cKO: F = 3.1330, p = 0.0906
3L	WT = 16, cKO = 12	One-way ANOVA	F = 6.8442, p = 0.0146
3M	WT = 16, cKO = 12	One-way ANOVA	F = 5.1671, p = 0.0315
3N	All conditions = 12	One-way ANOVA	F = 9.8878, p = 0.0047
4C	All conditions = 4	Unpaired Two-Tailed t-test	P value<br>Htr2c 0.0103251<br>Slc4a5 0.0404024

			Dnah6 0.0607404<br>Alx4 0.00450653<br>Ogn 0.279186<br>Dnali1 0.377612<br>Cdh3 0.024937<br>Cfap65 0.211217<br>Fmod 0.220558<br>Sostdc1 0.63636
4D	All conditions = 5	Unpaired Two-Tailed t-test	P value<br>CP 0.00608228<br>IZ 0.462969<br>VZ/SVZ 0.0340989
4E	All conditions = 4	Unpaired Two-Tailed t-test	Sting-sh vs Sting-sh + Alx4 P value<br>CP 0.0491381<br>IZ 0.674018<br>VZ/SVZ 0.041696
4F	All conditions = 3	Unpaired Two-Tailed t-test	Control vs CMA: t=5.593, df=4, p= 0.0050<br>Control vs cGAMP: t=2.808, df=4, p= 0.0484
4G	All conditions = 4	Unpaired Two-Tailed t-test	NF-κB-sh vs NF-κB-sh + Alx4 P value<br>cp 0.0448494<br>iz 0.49069<br>vz/svz 0.0415794
4I	All conditions = 4	One-way ANOVA	F (2, 9) = 26.73, P = 0.0002
4J	All conditions = 3	One-way ANOVA	F (2, 6) = 23.22, P = 0.0015
5B	All conditions = 3	Unpaired Two-Tailed t-test	p-NF-κB: p(0.5) = 0.0489407, p(2.5) = 0.0202157<br>alx4: p(0.5) =0.0287666 p(2.5) =0.0213823<br>tuj1: p(0.5) =0.0181925 p(2.5) =0.00551871
5C	All conditions = 4	Unpaired Two-Tailed t-test	P value<br>cp 0.119579<br>iz 0.478132<br>vz/svz 0.188647
5E	WT = 11, cKO = 10	One-way ANOVA	Call numbers: F = 1.1293, p= 0.3012<br>Call duration: F = 0.5225, p= 0.4786
5F	WT = 10, cKO = 10	One-way ANOVA	S1 vs E<br>WT: F = 65.2, p < 0.0001<br>cKO: F = 6.6532, p=0.0189<br>S1 vs S2<br>WT: F = 5.8067 p= 0.0269,<br>cKO: F = 6.4178, p=0.0208
7G	All conditions = 4	Unpaired Two-Tailed t-test	MAP2: t = 4.87791, df = 6, p = 0.00277209<br>SOX2: t= 3.32759, df=6, p = 0.0158541
7H	All conditions = 3	Unpaired Two-Tailed t-test	p-NF-κB: t = 1.87, df = 4, p = 0.135<br>SOX2: t=0.097, df=4, p = 0.93<br>TUJ1: t=1.26, df=4, p = 0.275
7J	All conditions = 3	Unpaired Two-Tailed t-test	PAX6: t = 7.345, df = 4, p = 0.00183<br>SOX2: t= 3.427, df=4, p = 0.0266<br>MAP2: t= 7.45, df=4, p = 0.00173

Extended Data Figures

Figure	Number of subjects	Test	Key comparisons
S1B	All conditions = 3	Unpaired Two-Tailed t-test	t=4.843, df=4, p= 0.0084
S2C	All conditions = 3	Unpaired Two-Tailed t-test	Sh1: t=13.20, df=4, p= 0.0002<br>Sh2: t=15.83, df=4, p < 0.0001
S2E	All conditions = 3	Unpaired Two-Tailed t-test	Sh1: t=3.414, df=4, p=0.0269<br>Sh2: t=5.502, df=4, p= 0.0053
S2F	All conditions = 4	Unpaired Two-Tailed t-test	Tbr2<br>sh1 : t=4.037, df=8, p= 0.0038<br>sh2 : t=6.504, df=8, p= 0.0002<br>Sox2<br>sh1 : t=2.527, df=6, p=0.0449<br>sh2 : t=6.753, df=6, p=0.0005
S3A	All conditions = 3	Unpaired Two-Tailed t-test	t=24.04, df=4, p< 0.0001
S3B	All conditions = 3	Unpaired Two-Tailed t-test	P value<br>cp 0.0166615<br>iz 0.446169<br>vzsvz 0.0483221
S3C	All conditions = 3	Unpaired Two-Tailed t-test	P value<br>cp 0.0073148<br>iz 0.331856<br>vzsvz 0.0246097
S4B	All conditions = 4	Unpaired Two-Tailed t-test	P value<br>cp 0.0286481<br>iz 0.823994<br>vz/svz 0.0195815
S4C	All conditions = 5	Unpaired Two-Tailed t-test	P value<br>STING< 0.0001<br>TUJ1 0.043969<br>PAX6 0.0277689
S4D	All conditions = 4	Unpaired Two-Tailed t-test	t=1.000, df=6, p=0.3559
S4F	All conditions = 10	Unpaired Two-Tailed t-test	t=4.728, df=18, p= 0.0002
S5B	All conditions = 6	Unpaired Two-Tailed t-test	P1: p= 0.0609575<br>adult : p= 0.0814403
S5E	All conditions = 12	One-way ANOVA	P value<br>S 0.228957<br>O 0.329457<br>N 0.0419735

S5F	All conditions = 12	One-way ANOVA	F=1.0079, p=0.3263
S6C	All conditions = 3	Unpaired Two-Tailed t-test	P value<br>Htr2c 0.0001740<br>Dnah6 0.000340<br>Slc4a5 0.002857<br>Alx4 0.0002475
S6D	All conditions = 3	Unpaired Two-Tailed t-test	P value<br>Htr2c-sh<br>CP 0.104971<br>IZ 0.120046<br>VZ/SVZ 0.589132<br>Dnah6-sh<br>CP 0.356784<br>IZ 0.598825<br>VZ/SVZ 0.134041<br>Slc4a5-sh<br>CP 0.393135<br>IZ 0.421405<br>VZ/SVZ 0.759858
S6J	All conditions = 3	Unpaired Two-Tailed t-test	SOX2: t=4.451, df=4, p= 0.0112<br>TBR2: t=4.904, df=4, p= 0.0080
S7A	All conditions = 4	Unpaired Two-Tailed t-test	P value<br>NF-κB 0.0180895<br>STAT6 0.0515844<br>TBK1 0.0859337<br>IRF3 0.328749
S7B	All conditions = 3	Unpaired Two-Tailed t-test	P value<br>IRF3 0.0074425<br>NF-κB 0.0102761<br>STAT6 0.00564391<br>TBK1 0.0253766<br>IKK2 0.00141629
S7C	All conditions = 3	Unpaired Two-Tailed t-test	P value<br>IRF3-sh<br>CP 0.179813<br>IZ 0.0913177<br>VZ/SVZ 0.815217<br>TBK1-sh<br>CP 0.6134<br>IZ 0.87104<br>VZ/SVZ 0.160817<br>STAT6-sh<br>CP 0.660759<br>IZ 0.637354<br>VZ/SVZ 0.687996
S7D	All conditions = 3	Unpaired Two-Tailed t-test	P value<br>CP 0.00218611

			IZ 0.262985<br>VZ/SVZ 0.0216966
S7F	All conditions = 4	Unpaired Two-Tailed t-test	P value<br>CP 0.0495611<br>IZ 0.738386<br>VZ/SVZ 0.0410266

Supplementary Figures and Figure legends

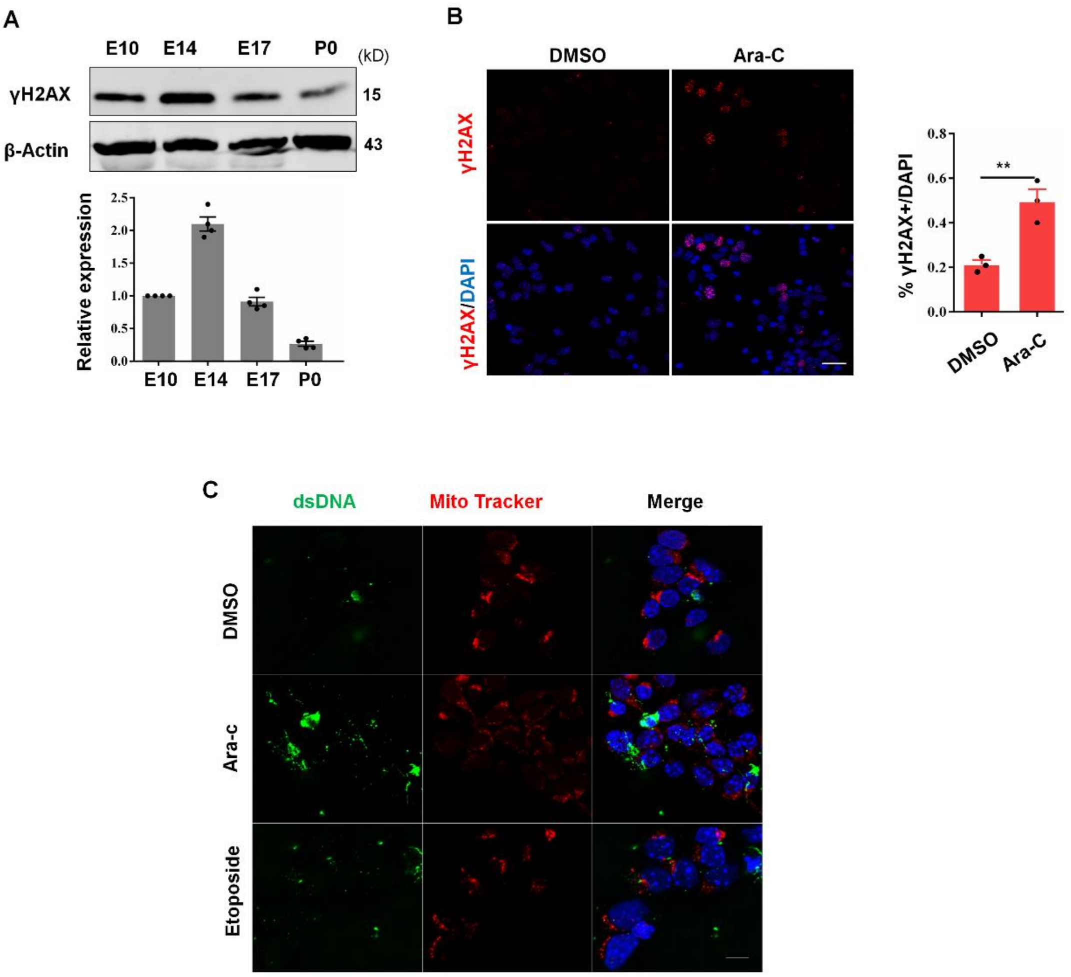

Figure. S1 DNA damage in NPCs related to Figure 1. (**A**)Western blot analysis of γH2AX protein levels in cerebral cortex of different developmental stages (E10, E14, E17, and P0) (*n*=3 experiments). (**B**) NPCs were isolated from E12.5 cerebral cortex and cultured for 1day. Then cells were treated with 1μM Ara-C for 8hr and stained for γH2AX and DAPI. Quantification of percentage of γH2AX^{+} cells in 9–15 fields randomly chosen from three independent experiments. Scale bar, 30μm. (**C**) NPCs cells were treated with 1μM Ara-C or 10μM Etoposide for 8hr and incubated with Mito Tracker for 30mins before stained for dsDNA and DAPI. Scale bar, 10μm. Error bars represent means ± SEM; two-tailed unpaired t-test, n.s., not significant, P<0.05 (*), P < 0.01(**) or P < 0.001(***).

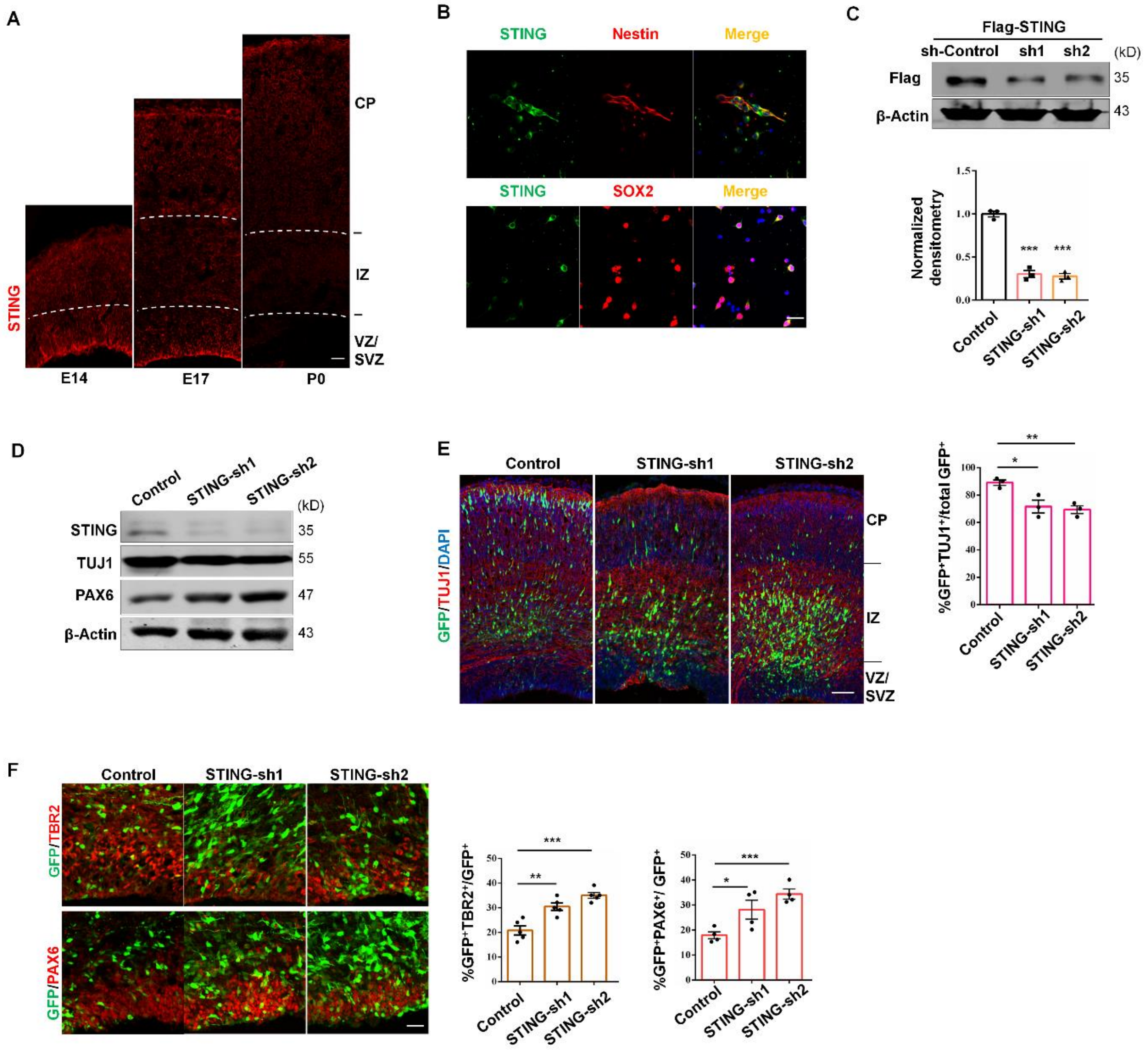

Figure. S2. STING is expressed in NPCs both in vitro and in vivo, and the development of cerebral cortex was affected by STING-shRNAs (**A**) Immunostaining for STING in the developing cortex. Embryonic brain sections were labeled with anti-STING antibody. STING expression decreased in the VZ/SVZ from E14 to P0 (n = 3 mice per period). Scale bar, 50 μm. (**B**) STING is colocalized with SOX2 and NESTIN in NPCs. The cells were isolated from the E12.5 cerebral cortex and cultured in proliferative medium for 1day (n = 4 experiments). Scale bars, 20 μm. (**C**) Co-expression of Flag-STING with STING knockdown plasmids in HEK293T cells. The efficiency of knockdown is analyzed by Western blot (n=3 experiments). (**D**) NPCs were infected with control or STING-shRNA lentivirus. The cells were infected for 2days before lysed for western blot of STING, TUJ1 and PAX6. (**E**) The control or STING knockdown plasmids were electroporated into the brain at E13 and brain sections were immunostained for TUJ1 at E16. The percentage of GFP and TUJ1 double-positive cells relative to the total GFP cells is shown as a bar graph (n=3 brains). Scale bars,

100 μm. (**F**) The percentage of GFP and PAX6 (or TBR2) double-positive progenitor cells is increased by STING knockdown. The control or STING knockdown plasmids were electroporated into the brain at E13 and brain sections were immunostained for PAX6 or TBR2 at E16. The percentage of GFP^+ $PAX6^+$ or GFP^+ $TBR2^+$ cells relative to the total GFP-positive cells in the VZ/SVZ is shown (n = 4 brains). Scale bars, 30 μm. Error bars represent means ± SEM; two-tailed unpaired t-test, n.s., not significant, P<0.05 (*), P < 0.01(**) or P < 0.001(***).

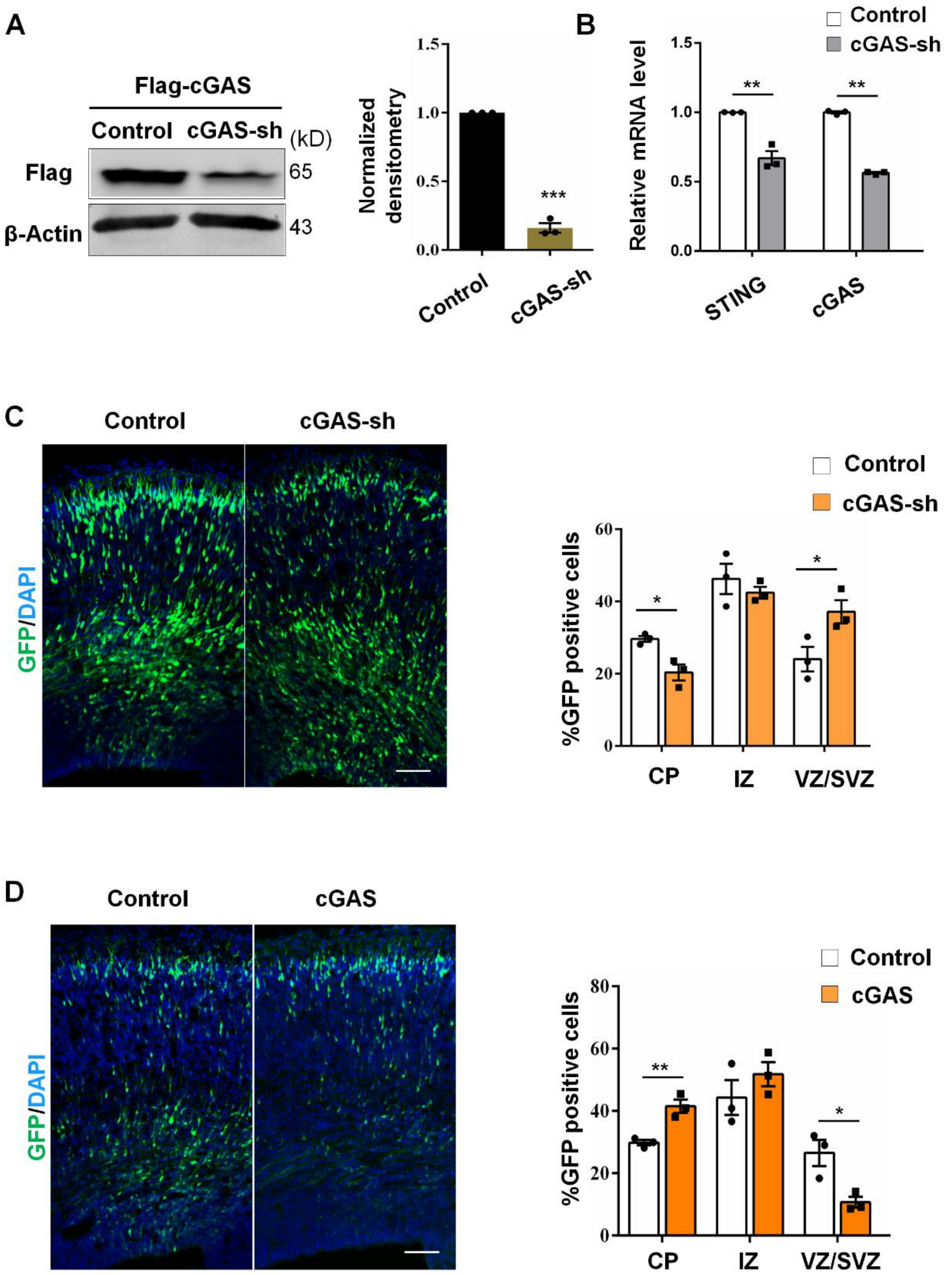

Figure. S3. Knockdown or overexpression of cGAS in NPCs, related to figure1. (**A**) Co-expression of Flag-cGAS with cGAS knockdown plasmid in HEK293T cells. The efficiency of knockdown is analyzed by western blot (n=3 experiments). (**B**) NPCs were infected with control or cGAS-shRNA lentivirus. The cells were infected for 2days before lysed for RT-qPCR of STING and cGAS. (**C**) cGAS knockdown results abnormal cell position in embryonic cerebral cortex. cGAS knockdown or control plasmid was electroporated into

brain of E13 and analyzed at E16. Bar graph shows the percentage of GFP-positive cells in each region (n=3 brains). Scale bar: 100 µm. (**D**) cGAS overexpression results in abnormal cell position in embryonic cerebral cortex. cGAS overexpression or control plasmid was electroporated into brain of E13.5 and analyzed at E16. (n=3 brains). Scale bar: 100 µm. Error bars represent means ± SEM; two-tailed unpaired t-test, n.s., not significant, P<0.05 (*), P < 0.01(**) or P < 0.001(***)

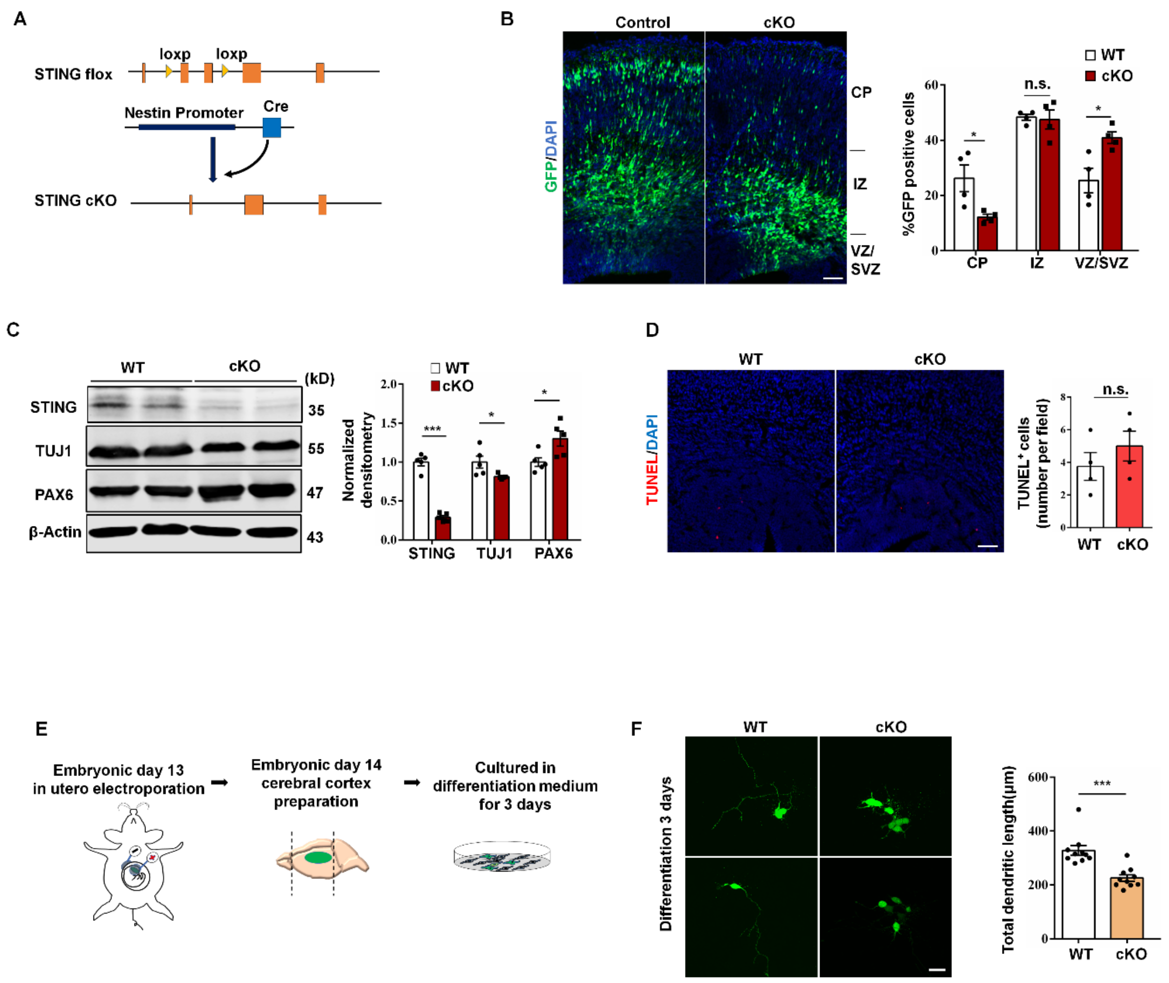

Figure. S4. STING deletion affects embryonic brain development in mice. (**A**) Schematic of the STING conditional knockout mice generation strategy. The STING gene was knocked out by NPCs-specific Cre recombinase splicing. (**B**) GFP-positive cell positioning is abnormal in STING ablated embryonic cerebral cortex. GFP plasmid was electroporated at E13.5 of STING$^{fl/fl}$ mice and STING^{cKO} mice, and the mouse brains were collected and analyzed at E16.5. Bar graph shows the percentage of GFP-positive cells in each region (n = 4 embryos from 3 different mothers). Scale bar, 100 µm. (**C**) Western blot analysis of STING, TUJ1 and PAX6 protein levels of cerebral cortex of E15 STING$^{fl/fl}$ and STING^{cKO} mice. Bar graph

shows quantification of normalized densitometry of STING, TUJ1 and PAX6. (*n*=5 brains). (**D**) E16 neocortex sections of $STING^{fl/fl}$ and $STING^{cKO}$ mice were subjected to TUNEL staining. Apoptosis levels were detected using the In Situ Cell Death Detection Kit (Roche). Graph shows the numbers of $TUNEL^{+}$ cells per field. Scale bar, 100 μm. (**E** and **F**) GFP plasmid was electroporated into the E13 brains of $STING^{fl/fl}$ or $STING^{cKO}$ mice. After 24 h, the electroporated brains were collected and the GFP^{+} cells were isolated and cultured for 3 days. Bar graph shows that the total dendritic length of neurons (n=10 cells from three samples). Scale bar, 15 μm. Error bars represent means ± SEM; two-tailed unpaired t-test, n.s., not significant, $P<0.05$ (*), $P < 0.01$(**) or $P < 0.001$(***).

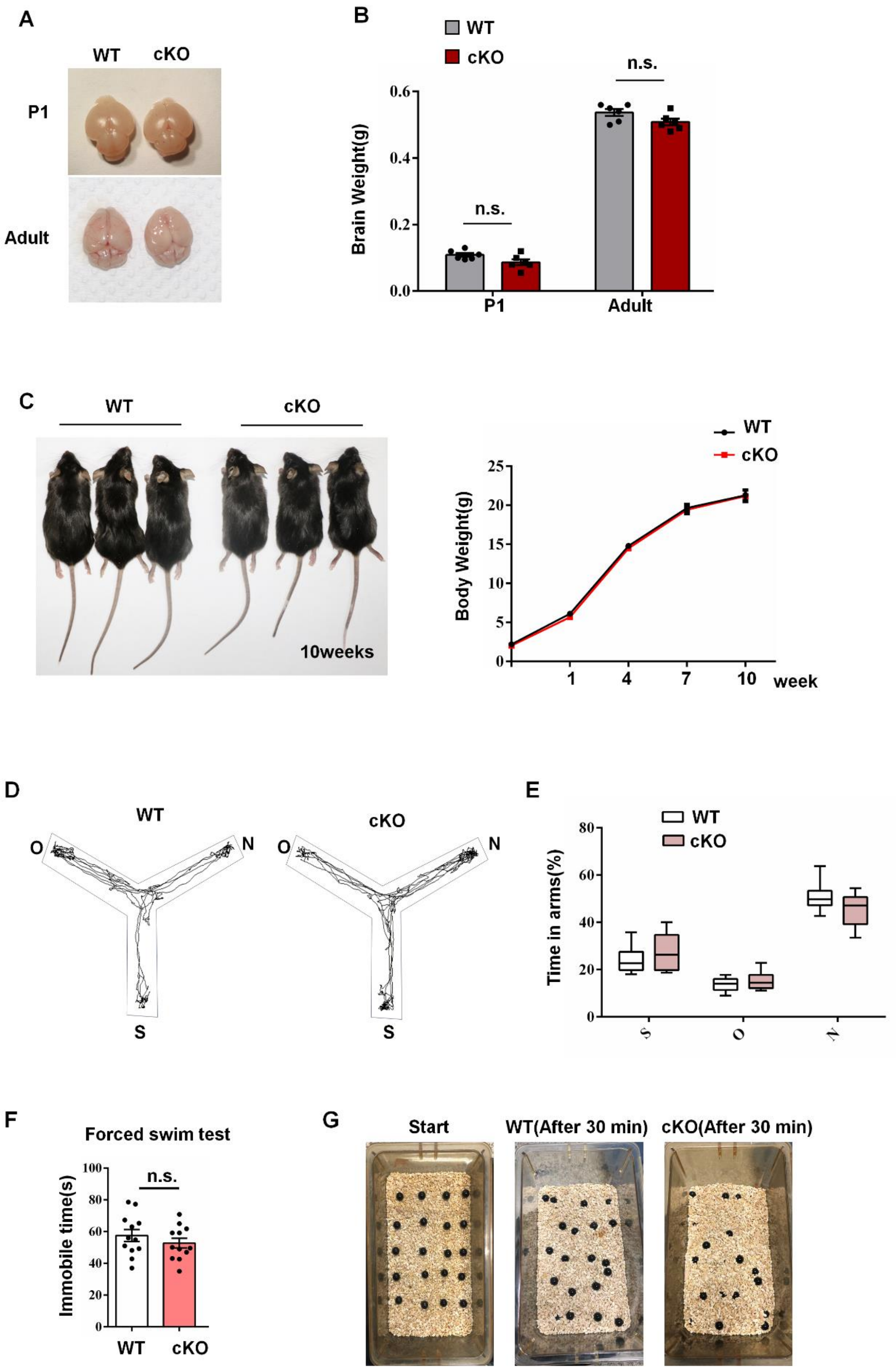

Figure. S5 CKO mice display ASD-like behaviors, related to figure3. (**A** and **B**) Brain weight of P1 and two-month-old STING^{cKO} and WT mice (n=6 brains). (**C**) Body weight STING^{cKO} and WT mice (n=12 mice). (**D**) Representative tracks from the Y-maze. (**E**) Time

spent in start, old and new arms of WT and cKO mice in Y-maze test (n=12). (**F**) The immobile time during the forced swim test. (**G**) Representative images of marble-burying behavior. Error bars represent means ± SEM; P values were calculated by two-tailed unpaired t-test (**B**, **C**) or one-way ANOVA with Tukey's post hoc (**E, F**), n.s., not significant, P<0.05 (*), P < 0.01(**) or P < 0.001(***).

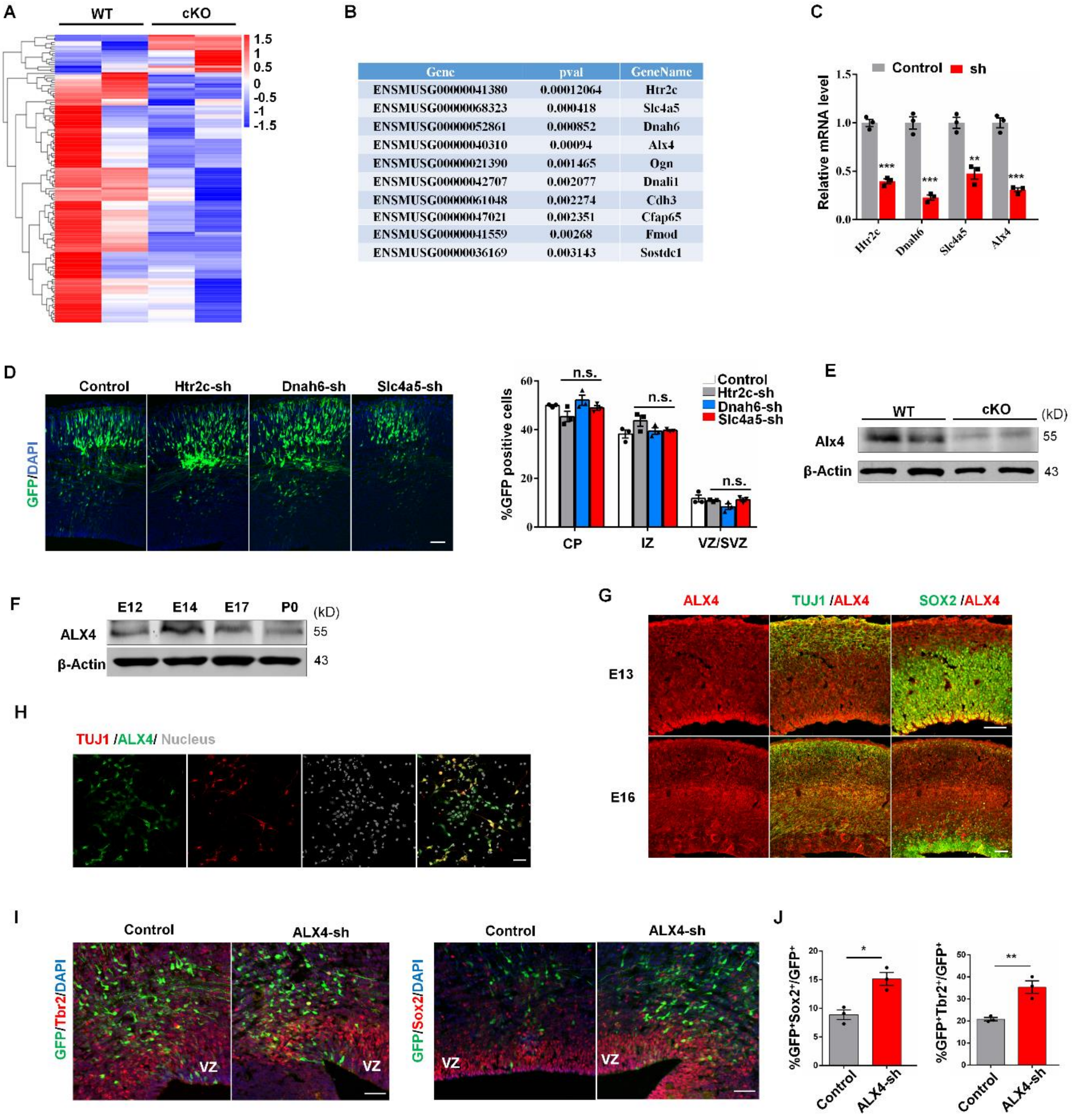

Gene	pval	GeneName
ENSMUSG00000041380	0.00012064	Htr2c
ENSMUSG00000068323	0.000418	Slc4a5
ENSMUSG00000052861	0.000852	Dnah6
ENSMUSG00000040310	0.00094	Alx4
ENSMUSG00000021390	0.001465	Ogn
ENSMUSG00000042707	0.002077	Dnali1
ENSMUSG00000061048	0.002274	Cdh3
ENSMUSG00000047021	0.002351	Cfap65
ENSMUSG00000041559	0.00268	Fmod
ENSMUSG00000036169	0.003143	Sostdc1

Figure. S6 ALX4 is downstream of STING in the developing cortex, related to figure4. (**A**) Heatmap analysis showed the gene profiling expression. Genes were selected by thresholds of p value <0.05 and log2FoldChange >=1 between WT and cKO. (**B**) List of top 10 genes that are downregulated when STING is depleted. (**C**) Knockdown efficiency of Htr2c, Dnah6, Slc4a5 or Alx4 by knockdown plasmid was analyzed in N2A cells (*n*=3 independent experiments). (**D**) Representative images of cortex sections which were

electroporated with control, Htr2c-shRNA, Dnah6-shRNA or Slc4a5-shRNA in utero at E13 and analyzed at E16 (n = 4 brains for all samples). Bar showed the percentage of GFP cells in the CP, IZ and VZ/SVZ (*n* = 4 brains for all samples). (**E**) Western blot analysis shows that Alx4 protein is decreased in E16 forebrain upon STING loss. (**F**) Western blot analysis of Alx4 protein levels in cerebral cortex of different developmental stages (E12, E14, E17, and P0). (**G**) Representative images of brain sections at E13 and E16 co-stained for ALX4 with TUJ1 or SOX2. Scale bars, 50 μm. (**H**) NPCs were isolated from E12.5 cerebral cortex and cultured for 2days. Then cells were co-stained with anti-Alx4 and anti-TUJ1 antibodies. Scale bar, 15mm. (**I** and **J**) The percentage of GFP and SOX2 (or TBR2) double-positive progenitor cells is increased by Alx4 knockdown. The control or Alx4 knockdown plasmids were electroporated into the brain at E13 and brain sections were immunostained for SOX2 or TBR2 at E16. The percentage of $GFP^{+}SOX2^{+}$ cells or $GFP^{+}TBR2^{+}$ relative to the total GFP-positive cells in the VZ/SVZ is shown (n = 3 brains). Scale bars, 50 μm. Error bars represent means ± SEM; two-tailed unpaired t-test, n.s., not significant, P<0.05 (*), P < 0.01(**) or P < 0.001(***).

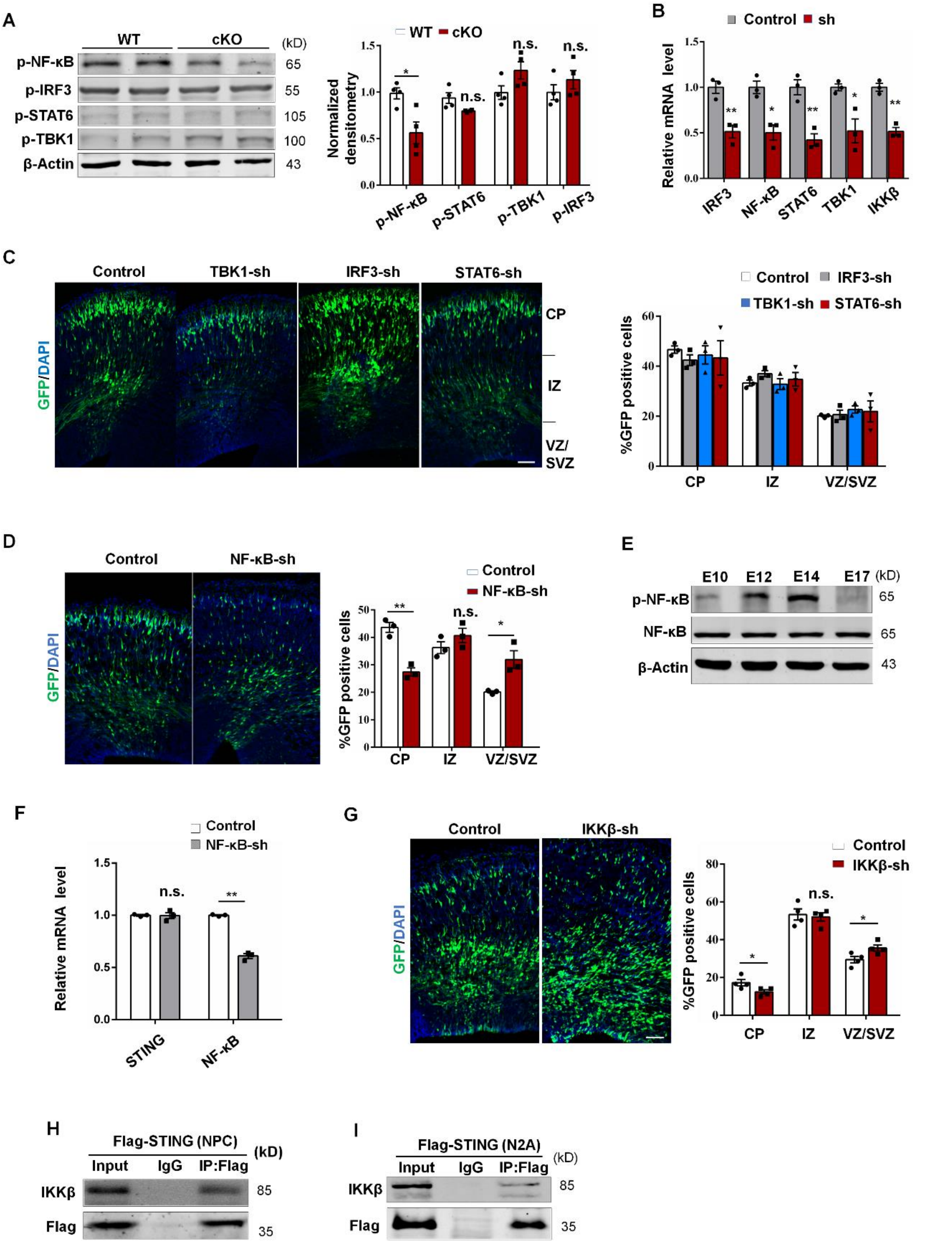

Figure. S7. STING regulates brain development by NF-κB signaling pathway, related to figure4. (**A**) Western blot analysis of p-NF-κB (p65), p-IRF3, p-STAT6 and p-TBK1 protein levels of cerebral cortex of E15 STING$^{fl/fl}$ and STING^{cKO} mice. Bar graph shows the quantification of normalized densitometry of p-NF-κB, p-IRF3, p-STAT6 and p-TBK1. (n=4 brains). (**B**) Knockdown efficiency of IRF3, NF-κB, STAT6, TBK1 or IKKβ was analyzed by knockdown plasmid in N2A cells (*n*=3 independent experiments). (**C**) Representative images

of cortex sections which were electroporated with Control, TBK1-shRNA, IRF3-shRNA or STAT6-shRNA in utero at E13 and analyzed at E16 (*n* = 3 brains for all samples). (**D**) Control or NF-κB (p65)-sh plasmids was electroporated into the brains at E13, and then the brains were harvested at E16. Bar graph shows the percentage of GFP cells in the CP, IZ and VZ/SVZ (n = 3 brains). Scale bar, 50μm. (**E**) Western blot analysis of p-NF-κB (p65) and NFκB (p65) protein levels in cerebral cortex of different developmental stages (E12, E14, E17, and P0). **(F**) NPCs were infected with control or NF-κB-shRNA lentivirus. The cells were infected for 2days before lysed for RT-qPCR of STING and NF-κB. **(G**) Representative images of cortex sections which were electroporated with control or IKKβ-sh at E13 and analyzed at E16. Bar graph shows the percentage of GFP cells in the CP, IZ and VZ/SVZ (*n* = 3 brains for all samples). (**H** and **I**) The interaction between STING and IKKβ was verified by co-IP experiments. Flag-STING-expressing lentivirus or plasmids were infected or transfected into NPCs (**H**) or N2A cells (**I**). Two days later, the infected or transfected cells were subjected to immunoprecipitation using flag beads. IKKβ and Flag-STING proteins were detected by western blotting with the antibodies indicated. Error bars represent means ± SEM; two-tailed unpaired t-test, n.s., not significant, $P<0.05$ (*), $P < 0.01$(**) or $P < 0.001$(***).
